# Synthetic biology and bioelectrochemical tools for electrogenetic system engineering

**DOI:** 10.1126/sciadv.abm5091

**Published:** 2022-05-04

**Authors:** Joshua M. Lawrence, Yutong Yin, Paolo Bombelli, Alberto Scarampi, Marko Storch, Laura T. Wey, Alicia Climent-Catala, PixCell iGEM Team, Geoff S. Baldwin, Danny O’Hare, Christopher J. Howe, Jenny Z. Zhang, Thomas E. Ouldridge, Rodrigo Ledesma-Amaro

**Affiliations:** 1 Department of Biochemistry, University of Cambridge, Cambridge, UK.; 2 Yusuf Hamied Department of Chemistry, University of Cambridge, Cambridge, UK.; 3 Department of Bioengineering, Imperial College London, London, UK.; 4 Department of Environmental Science and Policy, Università degli Studi di Milano, Milano, Italy.; 5 London DNA Foundry, Imperial College Translation and Innovation Hub, London, UK.; 6 Department of Chemistry, Imperial College London, London, UK.; 7 Department of Life Sciences, Imperial College London, London, UK.

## Abstract

Synthetic biology research and its industrial applications rely on deterministic spatiotemporal control of gene expression. Recently, electrochemical control of gene expression has been demonstrated in electrogenetic systems (redox-responsive promoters used alongside redox inducers and electrodes), allowing for the direct integration of electronics with biological processes. However, the use of electrogenetic systems is limited by poor activity, tunability, and standardization. In this work, we developed a strong, unidirectional, redox-responsive promoter before deriving a mutant promoter library with a spectrum of strengths. We constructed genetic circuits with these parts and demonstrated their activation by multiple classes of redox molecules. Last, we demonstrated electrochemical activation of gene expression under aerobic conditions using a novel, modular bioelectrochemical device. These genetic and electrochemical tools facilitate the design and improve the performance of electrogenetic systems. Furthermore, the genetic design strategies used can be applied to other redox-responsive promoters to further expand the available tools for electrogenetics.

## Teaser

A variety of genetic and electrochemical tools can be used to control the expression of genes using electricity.

## INTRODUCTION

Advances in synthetic biology have allowed for the development of a variety of genetic circuit-based devices used for sensing applications (*1*), drug and chemical production (*2*), and material synthesis (*3*). However, deterministic regulation of these systems is limited compared to the precise system control provided by electronic circuits. Integrating the control of electronic circuits with the biocatalytic activities of cells has the potential to create reliable and affordable devices for various biomedical and industrial applications (*4*).

One major method used to control biological systems is the use of inducible gene expression systems, which consist of a promoter and its cognate transcription factor that can either activate or repress the expression of genes in response to external stimuli (*5*). These systems have been used in molecular biology for the study of biomolecular systems in a variety of organisms (*6*–*8*). Inducible gene expression systems also serve as key tools in synthetic biology, used as inputs for genetic logic circuits (*9*) in microbial and cell-free biosensors (*10*, *11*), and as control systems for CRISPR-Cas9 genome editing (*12*), to name but a few applications.

**Fig. 1.**
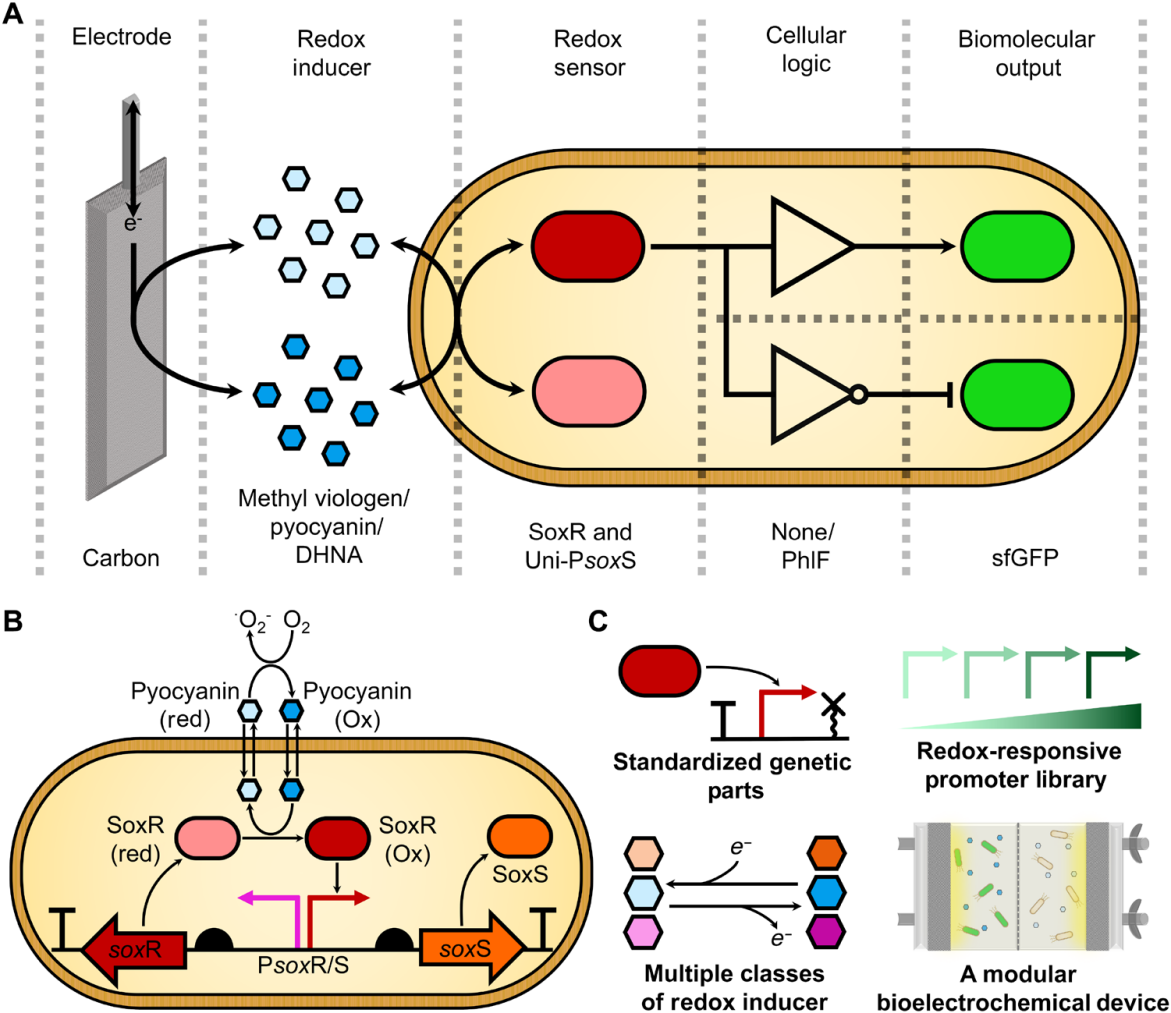
Electrochemical control of gene expression in electrogenetic systems. (**A**) Electrogenetic systems consist of a series of electrochemical and genetic modules. An electrode controls the redox state of a redox mediator that the cell responds to with a redox-sensitive transcription factor. Depending on the cellular logic encoded by the genetic architecture used, oxidation of the transcription factor will either activate or repress expression of the chosen gene of interest. The identity of specific components tested in this study is labeled beneath the schematic. (**B**) The native *sox*RS oxidative stress response system of *E. coli*. SoxR is constitutively expressed from the P*sox*R promoter. Oxidation of SoxR by oxidative stress–inducing redox molecules such as pyocyanin causes transcriptional activation of the P*sox*S promoter. This activation leads to SoxS expression, which activates transcription of numerous genes involved in the oxidative stress response. The redox dependency of P*sox*S allows for it to be used for electrochemical induction of gene expression. (**C**) An overview of the genetic and electrochemical tools developed in this study for use in electrogenetics systems.

While chemically inducible and light inducible (optogenetic) gene expression systems are the most popular, due to their strong activities, simplicity, and robustness (*13*, *14*), other inducible systems have been identified or engineered that respond to heat (*15*), magnetism (*16*), pressure (*17*), osmolarity (*18*), and salinity (*19*). Electrogenetics is a nascent field of research in which electrical and electrochemical stimuli are used to control biological processes, including gene expression (*4*, *20*). Electrogenetic systems consist of four components: an electrode, a redox inducer, a redox-transcription factor, and its cognate promoter (Fig. 1A). The electrode is held at a set potential that is used to oxidize or reduce the redox inducer, which is a cell-permeable electron mediator. The redox inducer in turn oxidizes or reduces the redox-transcription factor, which leads to activation or repression of gene expression from its cognate promoter.

Unlike other inducible gene expression systems, electrochemical control of gene expression allows for electronic and genetic circuits to be directly connected to one another. The ability to combine electronic controllers easily with cellular biocatalysts is expected to revolutionize bioelectronics, facilitating easy design of devices for biosensing, medical diagnostics and therapies, information processing, and energy conversion (*4*, *21*, *22*). Electrogenetic systems also have high spatiotemporal control compared to other widely used gene expression systems. They can be used to provide rapid oscillatory gene expression (*20*) and spatial gradients of gene expression across cells immobilized in hydrogels (*23*). Furthermore, in electrogenetic systems, gene expression can be tuned to a specific level simply by changing the electrode potential, which is more convenient than in chemically inducible systems where the addition or removal of inducers is required (*20*). They also avoid the phototoxicity and requirement for specific light conditions associated with optogenetic systems, as well as being better suited to larger culture volumes (such as in bioreactors) or implantable devices where light penetration can be an issue. Already, electrogenetic systems capable of performing digital-to-biological data storage (*24*) and electrically controlled in vivo insulin delivery (*25*) have been developed. Despite these advances, electrogenetics remains an emerging field, with the development of new systems being hindered by a lack of effective and standardized tools, whether they be electrochemical apparatus, redox inducers, or genetic parts. The use of the systems has also been limited because of many of them requiring anaerobic conditions (*20*) or complex cocultures (*26*). Oxidation of redox inducers by oxygen is common, which complicates accurate control of gene expression and can lead to the formation of cytotoxic reactive oxygen species under aerobic conditions.

In a pioneering study, Tschirhart *et al.* (*20*) first demonstrated electronic control of gene expression in bacteria by repurposing the *sox*RS oxidative stress response operon of *Escherichia coli* (Fig. 1B). In this system, a gold electrode was used, with the redox inducer pyocyanin oxidizing the SoxR transcription factor to activate the expression of transgenes placed downstream of the P*sox*S promoter. The precise spatiotemporal control of gene expression that has been demonstrated with SoxR-P*sox*S makes it a desirable electrochemically inducible gene expression system (*20*, *23*). However, the system has not yet been used to activate gene expression controllably under aerobic conditions due to pyocyanin’s reactivity with oxygen (*27*). The native P*sox*S promoter also exhibits a limited dynamic range (*DynR*, the fold change between the maximum activity and the basal activity) when compared to chemical and optogenetic inducible systems (*13*, *14*, *20*). The lack of a P*sox*S promoter library and the bidirectional nature of the promoter also prevent rational design and standardized assembly of genetic circuits (*9*, *28*).

In this work, we redesigned the P*sox*S promoter to facilitate stronger electrochemical induction of gene expression. A unidirectional form of the P*sox*S promoter was engineered with a greater dynamic range, allowing for simple construction of genetic circuits. We also created a library of P*sox*S promoters with a spectrum of activities and tested activation of genetic circuits using our promoters with several different classes of redox inducer. Electrochemical activation of gene expression was demonstrated under aerobic conditions using a novel, modular bioelectrochemical device designed for use in electrogenetic systems. Last, electrochemical tuning of gene expression was also demonstrated in a species of bacteria not previously used in electrogenetics research. The genetic and electrochemical tools we have developed (Fig. 1C), along with the modular framework used to construct these electrogenetic systems (Fig. 1A), will aid the design of future electrogenetic systems.

## RESULTS

### Redesigning the redox-responsive P*sox*S promoter

**Fig. 2.**
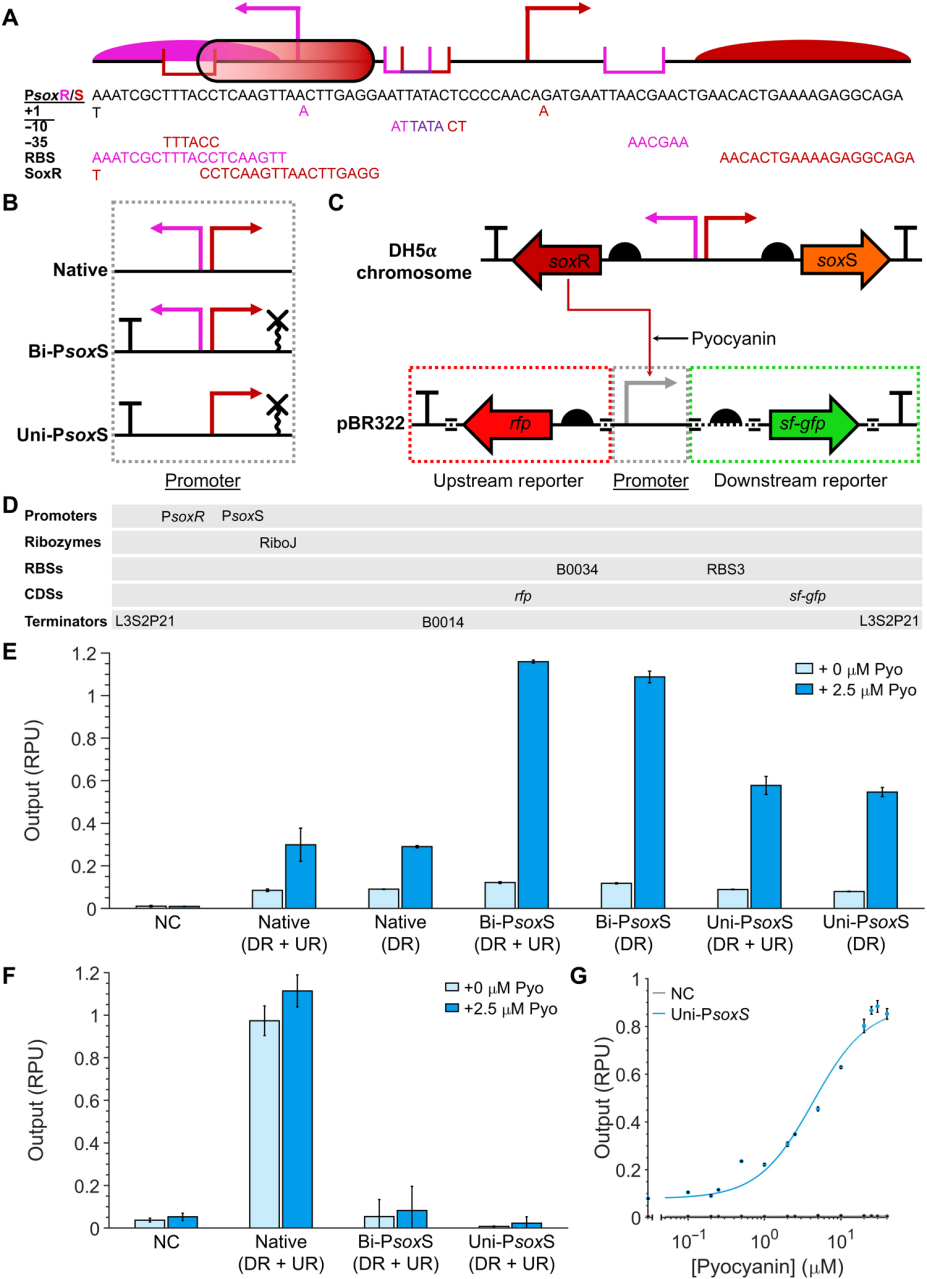
Screening the activity and directionality of mutant P*sox*S promoters. (**A**) The sequence of the native P*sox*R/S promoter, with key regulatory components of P*sox*R (pink), P*sox*S (red), and overlapping components (purple) labeled. TSS, transcription start site. (**B**) Architectures of native and engineered P*sox*S promoters tested. (**C**) Genetic circuit design for screening P*sox*S promoters for upstream and downstream transcriptional activity. Circuits can consist of both upstream reporter (UR) and downstream reporter (DR) or just the downstream reporter. Black dotted regions of the DNA represent BASIC (Biopart Assembly Standard for Idempotent Cloning) linkers. (**D**) List of genetic parts used. RBS, ribosome binding site; CDS, coding DNA sequence. (**E**) Downstream activity of engineered P*sox*S promoters with and without 2.5 μM pyocyanin. NC, negative control. (**F**) Upstream activity of engineered P*sox*S promoters with and without 2.5 μM pyocyanin. (**G**) The response function of the Uni-P*sox*S (downstream reporter) promoter with pyocyanin. Data points represent the mean from three biological replicates with error bars depicting SD (*n* = 3).

The bidirectional P*sox*R/S promoter lies at the core of the *sox*RS operon (Figs. 1B and 2A). The upstream P*sox*R promoter constitutively expresses the SoxR transcription factor, the Fe-S clusters of which can be oxidized by various redox-cycling drugs (*29*, *30*). SoxR binds to operator sites in the downstream P*sox*R promoter so that upon its oxidation, conformational changes in the protein bring these sites closer together, reducing the distance between the promoter −35 and −10 sites allowing for RNA polymerase recruitment and transcription of the gene for the SoxS regulatory protein (*31*).

The bidirectional nature of the P*sox*R/S makes it hard to use in genetic circuits, while its overlapping regulatory sequences make it difficult to generate mutant variants of the promoter (Fig. 2A) (*32*, *33*). To solve these problems, we have redesigned the P*sox*R/S promoter to obtain a unidirectional redox-responsive P*sox*S promoter. We created two variants of the native promoter: Bi-P*sox*S and Uni-P*sox*S. Both were designed with a standardized architecture of an upstream terminator, a spacer sequence, and a downstream RiboJ sequence (Fig. 2B) (*34*). These genetic elements were added to reduce promoter context dependency (*35*, *36*), while the terminator was additionally expected to remove upstream transcriptional activity and aid assembly of the promoter in multigene constructs. In Uni-P*sox*S, we additionally deleted the P*sox*R −35 regulatory sequence to enhance unidirectional promoter activity. We rationalized that removing P*sox*R activity would also enhance the activity of the promoter by favoring the recruitment of RNA polymerase in the P*sox*S direction.

We cloned native, Bi-P*sox*S, and Uni-P*sox*S promoter variants into genetic circuits capable of screening their activities through the measurement of fluorescent protein expression with a microplate reader. Downstream and upstream promoter outputs were measured through the stationary phase expression of superfolder green fluorescent protein (sfGFP) and red fluorescent protein (RFP), respectively (Fig. 2C). Fluorescence data were normalized by cell density before, before being divided by the same value recorded from a standardized reference plasmid (table S3) to calculate the promoter output in relative promoter units (RPUs) (see Materials and Methods) (*37*). This reference plasmid expressed reporters under the control of a J23101 promoter built with the same standardized architecture as Bi-P*sox*S and Uni-P*sox*S. Reporters were expressed under the control of strong synthetic ribosome binding sites (RBSs) (*28*) rather than the native RBS sequences (*33*) in all plasmids (Fig. 2D), and the gene circuits were assembled within a medium-high copy number vector [based on SEVA (Standard European Vector Architecture) pBR322] (*38*) to maximize recorded promoter outputs.

*E. coli* DH5α was transformed with either the assembled plasmids containing the promoter variant constructs (Fig. 2, B and C) or a negative control plasmid consisting of an empty pBR322 vector (table S3). *E. coli* DH5α contains an intact chromosomal copy of the *sox*RS operon constitutively expressing the SoxR transcription factor (Fig. 2C), allowing for the basal and induced activities of the P*sox*S promoter variants to be measured through the addition of the redox inducer pyocyanin. This screening method allowed for the high-throughput quantification and comparison of promoter outputs in microplates, allowing for the screening of many more variants than would be possible via electrochemical induction. A pyocyanin concentration of 2.5 μM was selected to maximize promoter outputs without incurring significant cytotoxicity, which we measured by cell growth inhibition (fig. S1).

All promoter variants displayed an induction of downstream promoter output in the presence of pyocyanin. However, both mutant variants displayed a significantly enhanced fold change between the uninduced and induced outputs compared to the native promoter (Fig. 2E). In constructs excluding the upstream reporter, the difference between the induced and uninduced downstream promoter outputs was 3.22-fold for the native promoter, compared to 9.20-fold and 6.86-fold for the Bi-P*sox*S and Uni-P*sox*S variants, respectively. Both mutant promoter variants we designed also lacked upstream promoter activity (Fig. 2F), facilitating their use in multigene devices without generating unwanted transcripts.

While the fold change of the Bi-P*sox*S promoter was higher than for the Uni-P*sox*S promoter, the latter has a significant 32.6% reduction in basal activity in the absence of pyocyanin than the former (Fig. 2E). Consequently, the Uni-P*sox*S variant was selected for use in future constructs. The downstream activity of the promoter was recorded in response to differing concentrations of pyocyanin and was fitted with a response function (*13*) (Fig. 2G and table S1). Response function curve fitting yielded a curve with cooperativity (*n*) of 1.22 and a sensitivity (*K*) of 4.29 μM. This *K* value is comparable to some of the highest performing chemically inducible gene expression systems (*13*).

Previous research has suggested that the P*sox*R promoter is repressed by SoxR (*39*). Furthermore, P*sox*S deletions in the spacer region between the −35 and −10 sites have been shown to convert the promoter from an activator to a repressor in certain strains of *E. coli* (*40*). With this in mind, we designed a unidirectional P*sox*R promoter variant (Uni-P*sox*R) and variants of the Uni-P*sox*S promoters with deletions between the −35 and −10 sites with the aim of creating promoters that are repressed rather than activated in the presence of pyocyanin. None of these exhibited significant fluorescence differences when pyocyanin was present (fig. S2) and were therefore not functional repressors in *E. coli* DH5α.

### Development of a redox-responsive promoter library

**Fig. 3.**
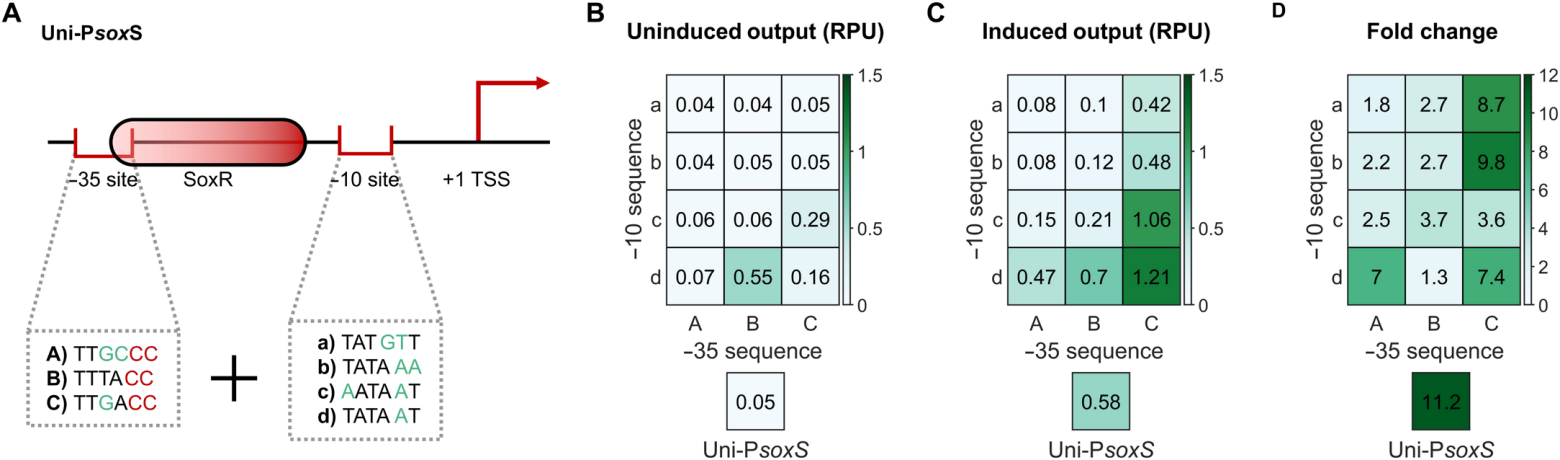
A Uni-P*sox*S promoter library. (**A**) Three −35 and four −10 site mutations were screened for tuning Uni-P*sox*S activity. −35 site mutations conserved nucleotides involved in SoxR binding. Promoters were tested using the genetic circuit in Fig. 2C. (**B**) Uninduced activity of the promoter library in the absence of pyocyanin. (**C**) Induced activity of the promoter library in the presence of 10 μM pyocyanin. (**D**) Fold change between induced and uninduced activities of the promoter library. Data points represent the mean from three biological replicates.

The deterministic design of complex genetic circuits requires accurate control of gene expression. Because of this, numerous promoter libraries have been generated for *E. coli*, including for both constitutive (*37*) and chemically inducible (*41*) promoters, allowing researchers to tune the expression of genes deterministically within genetic circuits. Here, we have developed a comparable library for Uni-P*sox*S through mutagenesis of the −35 and −10 sites. We selected three −35 sites (termed A to C) and four −10 sites (termed a to d) to test (Fig. 3A), which we expected to provide a range of promoter activities (*41*, *42*). The terminal cytosine nucleotides of the −35 site involved in SoxR binding were conserved in all variants (*32*). Uni-P*sox*S variants with different combinations of −35 and −10 sites were screened with the previously described downstream reporter construct (Fig. 2C), using a pyocyanin concentration of 10 μM to induce gene expression. This high concentration was selected to ensure that the response of any promoters with low sensitivity would be detected (Fig. 2F). Both the uninduced and induced outputs were measured, with the fold change between them being calculated to estimate the dynamic range. However, note that some promoters may have lower sensitivities and may not be fully induced in the presence of 10 μM pyocyanin, meaning that these fold changes may not be an accurate reflection of the dynamic range.

A range of outputs were observed among the Uni-P*sox*S mutants (Fig. 3, B to D, and fig. S3). While no mutations had enhanced fold change relative to unmutated Uni-P*sox*S, all mutants were inducible and had altered outputs. Some promoters with −10 sites a and b had significant reduction in both the basal and induced output of the promoter, making them preferable for the expression of toxic genes. Promoter mutants Bd, Cc, and Cd all had significantly higher basal and induced outputs, with mutant Cd providing the strongest output of any induced promoter at 1.21 RPU, which is comparable to the widely used p*Tet* and p*Lac* chemically inducible promoters (*37*). The development of this library allows for the sensitivity of gene expression to redox conditions to be rationally tuned in electrogenetic systems. For example, a strongly oxidizing condition will enhance gene expression from a weak mutant promoter to a lesser degree than it will for a strong mutant promoter. This additional level of control will aid the application of electrogenetic systems in synthetic biology, for example, for genetic logic gates, information-processing circuits, or metabolic pathways, which often require the differential expression of multiple genes at once.

### Enhancement and modulation of electrogenetic circuit responses

**Fig. 4.**
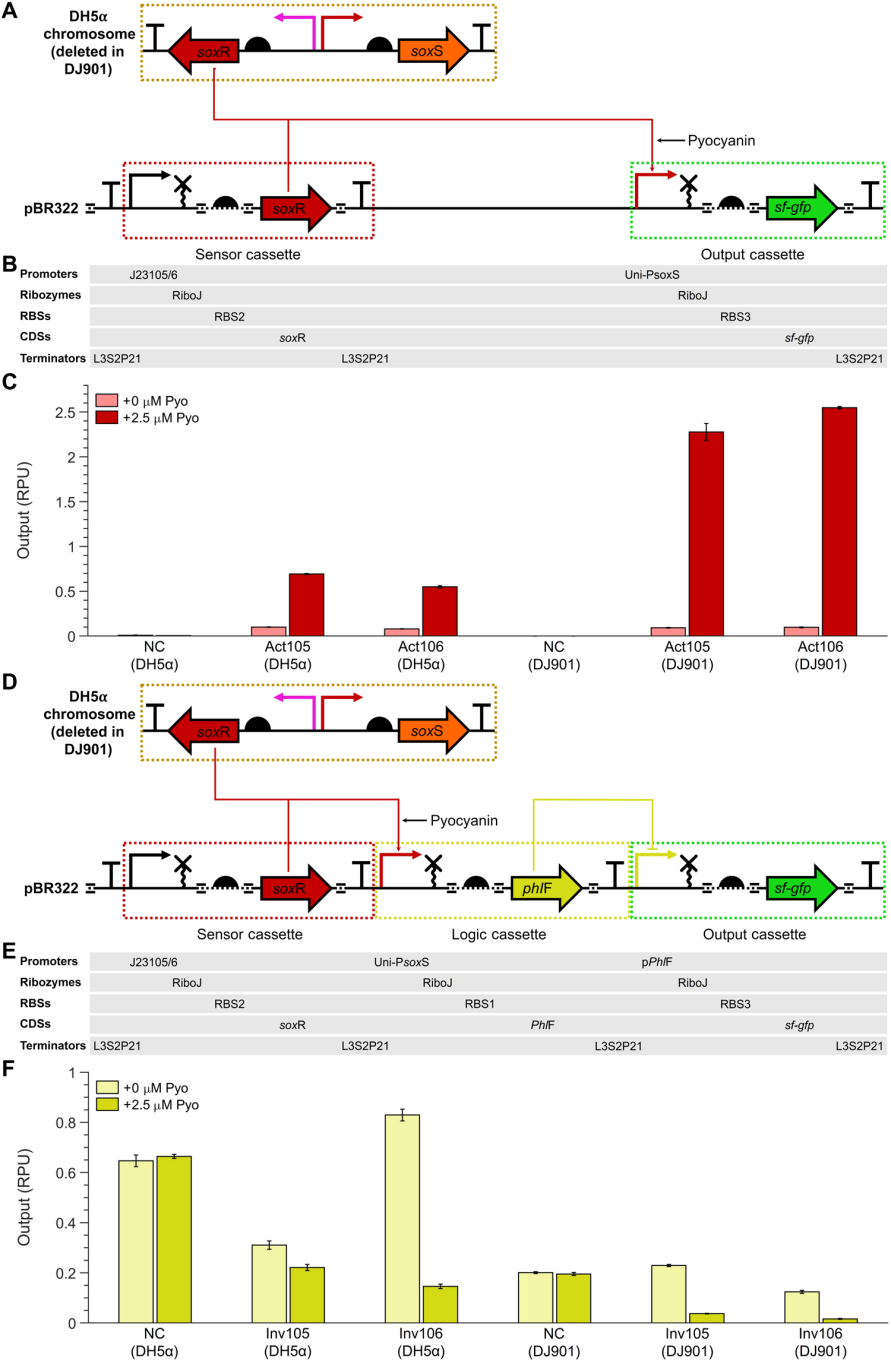
Performance of electrogenetic circuits in different *E. coli* strains. (**A**) Genetic circuit design for the activator device. A sensor cassette constitutively expressed SoxR, which, upon oxidation by pyocyanin, activates Uni-P*sox*S gene expression in the output cassette. In DH5α, the *sox*RS operon is found on the chromosome, while it is missing in the DJ901 deletion mutant. (**B**) List of genetic parts used in the activator device. (**C**) Activity of activator circuit with and without 2.5 μM pyocyanin. SoxR expression is controlled by J23105 in Act105 and J23106 in Act106. (**D**) Genetic circuit design for the inverter circuit. An additional logic cassette is incorporated so that SoxR oxidation by pyocyanin activates expression of PhlF, which in turn represses gene expression of the output cassette. (**E**) List of genetic parts used in the inverter circuit. (**F**) Activity of inverter circuit with and without 2.5 μM pyocyanin. SoxR expression is controlled by J23105 in Inv105 and J23106 in Inv106. Data points represent the mean from three biological replicates with error bars depicting SD (*n* = 3).

While the Uni-P*sox*S promoter is functional in *E. coli* DH5α due to its genomic copy of the *sox*RS (*43*), the low level of SoxR expression from the P*sox*R promoter (fig. S2) is not optimized for recombinant protein expression. To overcome this low SoxR expression, we assembled genetic circuits that not only expressed sfGFP under the control of Uni-P*sox*S in a “output cassette” but also contained a “sensor cassette” in which SoxR was expressed by a constitutive promoter (Fig. 4A). We developed two versions of these “activator” genetic circuits (Act105 and Act106) with SoxR expression being controlled by a different constitutive promoter in each (Fig. 4B). These constitutive promoters were created using the same standardized promoter design as Uni-P*sox*S (Fig. 2B) (*34*). We tested activator circuits in both DH5α and DJ901, a strain that contains a chromosomal deletion of the *sox*RS operon (*44*). This deletion prevents the strain from expressing antioxidant proteins (*39*) and multidrug efflux pumps (*45*), which we expected to enhance gene expression induction by pyocyanin, albeit at the expense of increased cytotoxicity (*20*).

Act105 and Act106 were screened for output in the absence and presence of 2.5 μM pyocyanin. A higher concentration was not used because of the susceptibility of DJ901 to oxidative stress. A negative control construct was also tested (table S3). All circuits demonstrated an enhanced output relative to the negative control, with circuits in DJ901 outperforming those in DH5α (Fig. 4C). Act106 (DJ901) had the best performance, with the highest induced output of 2.22 RPU with 26.3-fold activation being achieved upon addition of pyocyanin, both over twice that observed with Uni-P*sox*S alone in DH5α (Fig. 2E). Act105 and Act106 in DH5α did not provide improved performance relative to Uni-P*sox*S alone in DH5α. Furthermore, while the J23106 promoter used in Act106 is much stronger than the J23105 promoter used in Act105 (http://parts.igem.org), the two circuits had comparable outputs. This suggests that the activity of activator circuits in DH5α is relatively insensitive to SoxR expression.

Genetic circuit design also allows researchers to modulate the activity of inducible gene expression systems using logic gates. These systems can be simple NOT gates, also known as inverters, which simply invert the signal (change an activator to a repressor and vice versa) or can be more complex logic gates that integrate the signals from multiple input promoters into a single output (*9*). We constructed a second set of genetic circuits, which were similar in design to the activator circuits but included a “logic cassette” in addition to output and sensor cassettes (Fig. 4D). This logic cassette consisted of a PhlF repressor (*13*) being expressed under the control of Uni-p*Sox*S, with sfGFP being expressed from its cognate P*phl*F promoter (Fig. 4E). This logic cassette is a NOT gate, allowing us to repress rather than activate Uni-P*sox*S activity in response to an oxidized redox inducer such as pyocyanin. We therefore termed these “inverter” circuits.

Inverter circuits were tested in the same manner as the activator circuits. The negative control construct consisted solely of the logic and output cassettes but with the Uni-P*sox*S promoter in the logic cassette replaced with a constitutive promoter. All inverter circuits exhibited a repression of output in the presence of pyocyanin but to differing extents (Fig. 4F). The outputs of both circuits in DJ901 were very low, with the uninduced state having an output comparable to the basal activity of the activator circuits in the same strain. By comparison, the inverter circuit outputs in DH5α were significantly higher. While this could partially be attributed to the increased strength of Uni-P*sox*S output in DJ901, the reduced output of the negative control in this strain also suggests a differential activity of P*phl*F output between the two strains. In addition to the *sox*RS deletion in DJ901, DH5α also contains numerous genomic deletions not found in DJ901, which could contribute to this effect (*43*, *44*). Inv106 in DH5α produced the best response, with the highest uninduced output of 0.89 RPU, and a 5.69-fold repression being achieved upon addition of pyocyanin. By successfully demonstrating conversion of the SoxR-P*sox*S system into a repressor, we have expanded the applications of electrogenetic systems that use it.

The portability of the electrogenetic circuits was tested by screening the activity of the Act106 circuit in additional strains of *E. coli* [BL21(DE3) and MDS42] and in the fast-growing bacterium *Vibrio natriegens* [wild type (WT)] (fig. S4) (*46*). The circuit exhibited activation in the presence of pyocyanin in all these organisms, with 20.4-fold activation in BL21(DE3) and 17.3-fold activation in *V. natriegens* upon addition of 2.5 μM pyocyanin. MDS42 exhibited a lower 1.3-fold activation in the presence of 2.5 μM pyocyanin; however, this increased to a 3.4-fold activation at 10 μM pyocyanin. BL21(DE3) output instead decreased at 10 μM, potentially due to high toxicity. This demonstrates that the inducible promoter system is compatible with multiple strains and species of bacteria; however, the sensitivity of the circuits to redox inducers may differ between hosts.

### Screening for SoxR redox inducers

When designing an electrogenetic system, the choice of redox inducer is an important consideration. The membrane permeability of redox inducers will differ between organisms, while their midpoint potential and electron transfer chemistry will determine their effectiveness and their propensity to partake in cytotoxic or deleterious side reactions such as oxygen reactivity (*27*). Many organisms are also capable of synthesizing their own redox inducers, either naturally (*22*) or through genetic engineering (*27*), which can allow for electrogenetic systems to be created without the need for exogenous redox inducers.

**Fig. 5.**
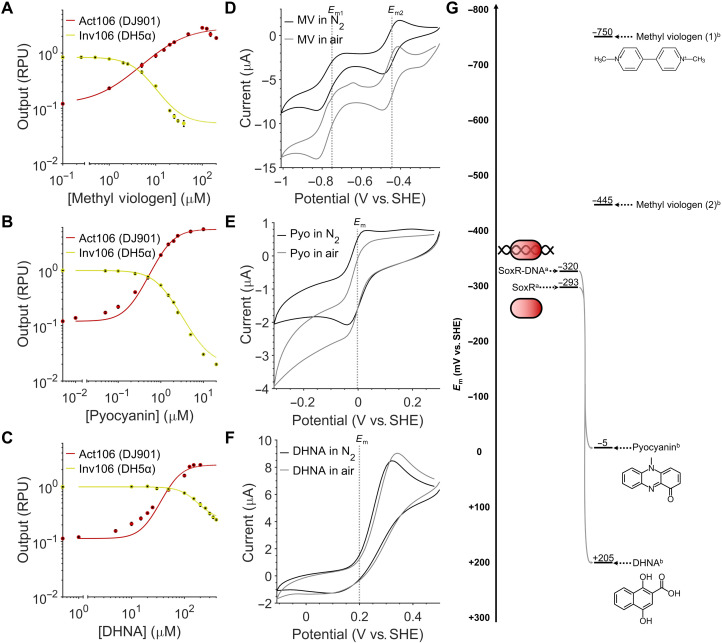
Electrogenetic circuit activation by diverse redox inducers. (**A** to **C**) The response function of activator Act106 (DJ901) and inverter Inv105 (DH5α) circuits to methyl viologen, pyocyanin, and DHNA. Data points represent the mean from three biological replicates with error bars depicting SD (*n* = 3). (**D** to **F**) Cyclic voltammograms of methyl viologen, pyocyanin, and DHNA recorded under aerobic (gray lines) and anaerobic (black line) conditions. LB medium was used as electrolyte with 1 mM methyl viologen, 100 μM pyocyanin, or 1 mM DHNA. The third cyclic voltammetry scan was recorded for each using a scan rate of 10 mV/s. Voltammograms were recorded both in air and in electrolyte purged with N_2_ gas for 30 min (to remove oxygen) before measurements were recorded with an N_2_ gas stream being maintained in the headspace. (**G**) Chemical structures and redox midpoint potentials (*E*_m_) of redox inducers methyl viologen, pyocyanin, and DHNA. Gray arrows indicate the possibility of direct oxidation of SoxR by compounds. *E*_m_ values from (a) Kobayashi *et al.* (*29*) and (b) this study.

The ability of SoxR to be oxidized by different classes of redox inducers provides researchers with flexibility when designing electrogenetic systems (*30*). We demonstrated this flexibility by testing the response of our activator and inverter electrogenetic circuits with five different redox inducers: pyocyanin (a natural phenazine), methyl viologen (a synthetic viologen), 1,4-dihydroxy-2-naphthoic acid (DHNA) (a natural quinone), riboflavin (a natural flavin), and hydrogen peroxide (Fig. 5G). All of these acted as redox inducers with the Act106 (DJ901) electrogenetic circuit apart from riboflavin and hydrogen peroxide (Fig. 5, A to C, and figs. S5 and S6). The lack of induction by hydrogen peroxide supports previous research that SoxR primarily responds to superoxide-generating, redox-cycling drugs (*47*). The instability of superoxide made it hard to test the response of circuits to superoxide alone; however, previous results have shown that *E. coli* SoxR is relatively insensitive to oxidation by superoxide (*48*, *49*).

To test whether the inactivity of riboflavin was caused by poor membrane permeability, cLogD values were calculated for each of the organic redox inducers (*27*) at physiologically relevant pH values (*50*) (fig. S7). cLogD predictions of greater than 0.5 for molecules with the size of pyocyanin, DHNA, or methyl viologen, or of greater than 1.7 for molecules with the size of riboflavin, mean that their probability of membrane permeability is over 50% (*51*). Oxidized and reduced pyocyanin and reduced methyl viologen met this condition, indicating a high membrane permeability. Oxidized methyl viologen had a highly negative cLogD, suggesting that it cannot permeate the membrane, which has previously been proven experimentally (*52*). This suggests that methyl viologen must be reduced by components of the medium or the plasma membrane to permeate the cell. cLogD values for DHNA were below the 0.5 threshold but were sufficiently high for an ~25% probability of membrane permeability (*51*), with the molecules previously being found to permeate bacterial membranes (*53*). Riboflavin had strongly negative cLogD values, which, alongside previous research demonstrating that *E. coli* lacks riboflavin transporters (*54*), could explain its lack of activity.

Response functions and growth curves were obtained with different redox inducers to identify the optimal inducer concentrations that would provide maximal induction without significant cytotoxicity. The response function of pyocyanin provided the highest *DynR* of 46.06 for Act106 (DJ901) and 49.97 for Inv106 (DH5α) (table S1). These values are comparable with some of the best performing chemically (between 100 and 2100) (*13*) and optogenetic (between 13.7 and 60.9) (*14*) inducible gene expression systems available. The maximum output for Act106 (DJ901) was also 4.82 RPU, which is larger than any of the high-performance, plasmid-based, “Marionette” chemically inducible gene expression systems that were produced by directed evolution (*13*). By comparison, methyl viologen and DHNA provide a lower but still workable *DynR* to pyocyanin (Fig. 5, A to C, and table S1). This improved ability of phenazines to activate the SoxR-P*sox*S system in comparison to viologens and quinones has been reported previously (*49*).

Cytotoxicity of the redox inducers varied (fig. S8). Methyl viologen was the most cytotoxic inducer, providing significant growth inhibition to both Act106 (DJ901) and Inv105 (DH5α) from concentrations of 1 μM. This toxicity is attributable to both its superoxide-generating ability and its synthetic nature, preventing it from being degraded by cells (*55*). As expected, pyocyanin was much more cytotoxic to Act106 (DJ901) due to the deletion of the entire *sox*RS operon in the strain (*44*), with cell growth being almost completely inhibited at 10 μM with Inv106 (DH5α) exhibiting a <20% growth inhibition at the same concentration. DHNA was only significantly cytotoxic to Inv106 (DH5α), which can likely be attributed to differences in the resistance of their respective background strains.

We also tested the performance and cytotoxicity of the Inv106 (DH5α) in M9 minimal medium using pyocyanin as a redox inducer, as previous results have suggested that minimal medium may provide improved fold changes with the SoxR-P*sox*S system (*20*) (fig. S9A and table S1). While the value of *K* is much smaller under this condition (0.09 μM in M9 versus 0.57 μM in LB), this improved sensitivity is achieved at the expense of a reduced *DynR* (49.97 in LB versus 38.15 in M9). Little difference in the cytotoxicity of pyocyanin in the two media was observed (figs. S8E and S9B).

Cyclic voltammetry was performed on the successful redox inducers pyocyanin, methyl viologen, and DHNA to determine better their electrochemical properties and the mechanism by which they oxidize SoxR (Fig. 5, D to F). Midpoint potentials (*E*_m_), the potential at which a 50% of the inducer is expected to be oxidized and 50% reduced, were determined from these voltammograms (Fig. 5G). If an *E*_m_ value of an inducer is more positive than the *E*_m_ of SoxR at −320 mV versus SHE (Standard Hydrogen Electrode) (in millivolts henceforth) when bound to DNA (*29*), then that redox inducer is theoretically capable of directly oxidizing the transcription factor. The −5-mV *E*_m_ of pyocyanin and +205-mV *E*_m_ of DHNA suggest that they are both capable of directly oxidizing SoxR and activating P*sox*S expression in the process. Direct oxidation of SoxR by phenazines and quinones has been described previously (*49*). In comparison, both the −750- and −445-mV *E*_m_ values of methyl viologen demonstrate that it is incapable of directly oxidizing SoxR and must therefore activate P*sox*S expression by an indirect mechanism. Previous research has found that SoxR oxidation by methyl viologen cannot be attributed to superoxide generation (*49*), suggesting that instead the redox inducer oxidizes SoxR by some other mechanism.

Voltammograms recorded in air (aerobic) and under N_2_-purging (anaerobic) conditions were also compared to determine the extent of oxygen reduction by these redox inducers. The relative insensitivity of *E. coli* SoxR to superoxide (*29*, *49*) and hydrogen peroxide (*47*) means that oxygen reduction by redox inducers is not expected to activate expression of genes downstream of P*sox*S but will instead limit the ability for the redox inducer to be fully reduced by an electrode. While cyclic voltammograms of DHNA showed no shift in current between scans recorded in air and under N_2_ purging, voltammograms of pyocyanin and methyl viologen displayed diminished oxidation (forward) peaks and altered reduction (backward) peaks in air (Fig. 5, D to F). This matches previous literature that describes the ability of pyocyanin and methyl viologen to generate reactive oxygen species (*56*). Current shifts were also observed in voltammograms recorded in LB in the absence of redox inducers, with a distinctive reduction peak being observed at −439 mV (fig. S10), suggesting that this is the peak of oxygen reduction.

With these results, we demonstrate the activation of the SoxR-P*sox*S system by various classes of redox inducers across a wide range of *E*_m_ values. Phenazines such as pyocyanin and quinones such as DHNA have an additional advantage in that they can be naturally synthesized. *E. coli* naturally synthesizes DHNA (*57*) and has also been genetic engineered to produce pyocyanin using genes from the *phz* operon of *Pseudomonas* (*58*). Other electrogenic bacteria are also known to produce a variety of other electron mediators that could also act as redox inducers (*22*).

### Electrochemical control of gene expression under aerobic conditions

Previous electrogenetic systems have used bespoke bioelectrochemical cells and devices to perform electrochemical control of gene expression (*20*, *23*–*26*). While functional, these ad hoc approaches lack standardization, preventing their use in different electrogenetic systems and hindering reproducibility between research groups. We have therefore assembled a bioelectrochemical device specifically designed for use in electrogenetic systems.

**Fig. 6.**
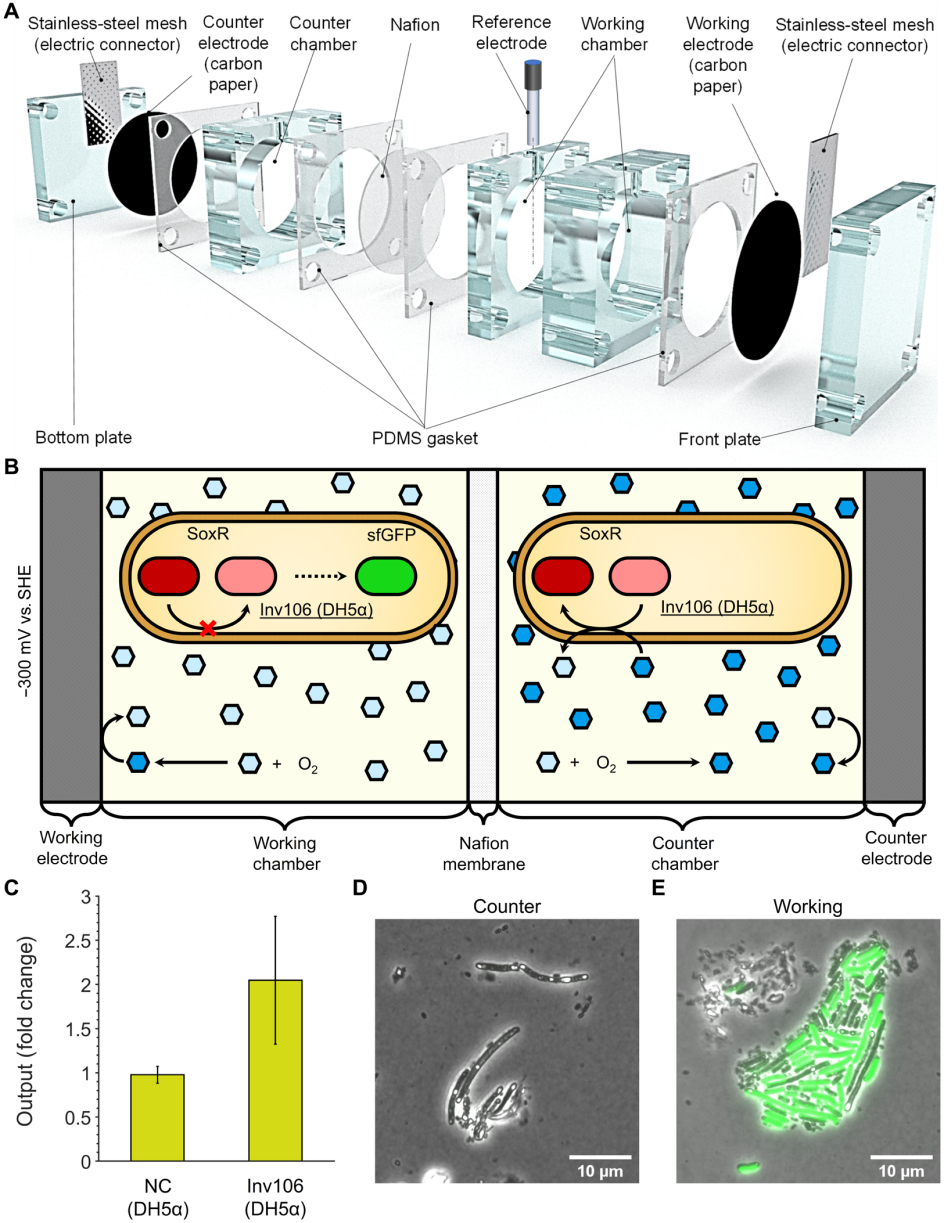
Electrochemical activation of gene expression under aerobic conditions. (**A**) Diagram of the modular bioelectrochemical device developed for performing electrochemical induction of gene expression. (**B**) Schematic of device operation for electrochemical activation of gene expression. The working electrode was held at a potential of −300 mV versus SHE to reduce pyocyanin, preventing oxidation of SoxR in Inv106 (DH5α) cells and thereby activating expression of sfGFP in the anodic chamber. (**C**) Gene expression change in cells between the working and counter chambers measured as a fold change. Data points represent the mean from three biological replicates with error bars depicting SD (*n* = 3). (**D** and **E**) Confocal fluorescence micrographs of Inv106 (DH5α) grown in the cathodic and anodic chambers and then immobilized on agarose pads during imaging. sfGFP fluorescence (λ_excitation_ = 490 nm) in green is overlaid on bright-field images.

This device consists of a stack of acrylic blocks and polydimethylsiloxane (PDMS) gaskets with cylindrical cavities for fastening with stainless steel screws. Electrically conductive materials serve as working and counter electrode materials with solid acrylic blocks placed at each end of the device. A Nafion membrane placed at the middle of the stack delimits the working and counter chambers, with magnetic stir bars providing mixing in each chamber (Fig. 6A and fig. S11). The modular nature of the device allows for it to be reconfigured for use with different electrode materials or chamber volumes, meaning that the device can be optimized for different applications. The system can be operated in two-, three-, or four-electrode mode by introducing one or more reference electrodes. In this study, a three-electrode setup was used with carbon paper working and counter electrodes and a Ag/AgCl reference electrode, with each chamber having a volume of 30 ml.

With this device, we performed electrochemical activation of gene expression under aerobic conditions, using pyocyanin as a redox inducer due to its maximal activation of the SoxR-P*sox*S system (Fig. 5B), lack of cytotoxicity with Inv106 (DH5α) (fig. S8E), and its previously recorded long-term stability in bioelectrochemical systems in comparison to quinone (*27*). Inv106 (DH5α) cells (Fig. 4D) were grown in both chambers of the device in LB electrolyte supplemented with 10 μM pyocyanin under a pyocyanin-reducing applied bias potential of −300 mV at the working electrode. If the rate of pyocyanin reduction by the working electrode exceeds the rate of pyocyanin oxidation by oxygen, then sfGFP expression is activated in cells within the working chamber by maintaining SoxR in a more reduced state. Alternatively, in the counter chamber, pyocyanin should be maintained in an oxidized state by the electrode and oxygen, leading to SoxR activation and suppression of sfGFP expression (Fig. 6B).

Under these conditions, Inv106 (DH5α) exhibited a 2.27-fold increase in sfGFP expression in the working chamber relative to the counter chamber, whereas the corresponding negative control (table S3) construct exhibited no significant difference in sfGFP expression between the two chambers (Fig. 6C and fig. S12). This fold change closely matches those reported for a different *E. coli* electrogenetic system under aerobic conditions (*26*), despite the SoxR-P*sox*S here allowing for a much simpler electrogenetic system without the use of specialized gold-binding strains, a coculture or minimal medium.

The fold change observed for electrochemical induction of Inv106 (DH5α) was much smaller than that recorded in dose-response measurements (Fig. 5F), suggesting that complete reduction of pyocyanin in the anodic chamber was not achieved. Confocal fluorescence microscopy of Inv106 (DH5α) cells from the anodic and cathodic chamber also revealed that while cells in the anodic chamber clearly exhibited much higher sfGFP fluorescence, some cells appeared to have reduced fluorescence, suggesting certain heterogeneity within the population (Fig. 6, D and E, and fig. S13). Regardless, these results demonstrate the first example of electrochemical activation of gene expression under reducing conditions and a proof of concept for electrochemical control of gene expression using the SoxR-P*sox*S system under aerobic conditions.

### Electrochemical tuning of gene expression in *V. natriegens*

A major advantage of electrogenetic systems is that gene expression can be specifically tuned by altering the redox state of the solution by changing the working electrode potential. This has previously been demonstrated in *E. coli* using an anaerobic electrogenetic system in which the response of the cells to a redox inducer is modulated by the relative concentration of ferricyanide (oxidized) and ferrocyanide (reduced) (*20*). We performed similar experiments using our genetic circuits and bioelectrochemical device with the bacteria *V. natriegens*, an emerging chassis for molecular and synthetic biology research.

Electrochemical tuning of sfGFP expression in Act106 (*V. natriegens*) cells was performed in the working electrode chamber of the bioelectrochemical device. LBv2 medium was used as the electrolyte and was supplemented with 10 μM pyocyanin and 5 mM ferrocyanide. A specific working electrode potential was applied for 30 min, after which cells were incubated for 4 hours, and sfGFP fluorescence was recorded. A strongly reducing potential (−300 mV) was applied to fully reduce the redox species, preventing SoxR oxidation and providing minimal sfGFP expression (fig. S14A). A strongly oxidizing (+600 mV) was applied to fully oxidize the redox species, ensuring complete SoxR oxidation and maximal sfGFP expression (fig. S14B). To demonstrate electrochemical tuning of gene expression, an intermediate potential (+450 mV) was applied to partially oxidize the redox species, in turn, only providing partial oxidation of SoxR for an intermediate level of sfGFP expression.

For all applied working electrode, potentials tested currents stabilized within minutes of the potential first being applied (fig. S14C). Normalized sfGFP fluorescence was 4.3-fold higher with the application of a +450 mV potential and 11.2-fold higher with the application of a + 600 mV potential when compared to the −300 mV condition (fig. S14D). These results clearly demonstrate tuning of gene expression relative to an applied electrode potential. Furthermore, these results also demonstrate the functionality of the genetic and electrochemical tools developed in this study in a bacterial species other than *E. coli*. Gene expression was also shown to positively correlate with the charge transferred at the electrode surface (fig. S14E), as has been shown in previous studies (*20*). This shows that gene expression can be tuned by controlling the electrochemical conditions in a manner similar to the response curves recorded with increasing concentrations of redox inducer (Fig. 5, A to C). It is also possible that the magnitude of the activation could be enhanced in future experiments through a longer application of the working electrode potential (*20*).

## DISCUSSION

Chemical and optogenetic inducible gene expression systems have been widely applied in molecular biology research and are being used in various biotechnological devices. While providing effective spatiotemporal control (*20*, *23*) and improved integration in bioelectronic devices (*4*), the use of electrochemical inducible gene expression systems has been limited because of their reduced performance and dependence on anaerobic conditions or cocultures. Our results demonstrate significant improvements to the performance of the SoxR-P*sox*S electrochemically inducible gene expression system, providing biological responses comparable to commonly used chemically inducible systems such as the AraC and LasR systems (*41*). A library of redox-responsive promoters allows for rational design of electrogenetic circuits (*9*), and a proof of concept for electrochemical gene activation under aerobic conditions is also presented. These promoters can be used to improve a previously developed eCRISPR system for multiplexed control of expression of multiple genes (*22*) and facilitate the construction of complex multilayered logic devices (*9*).

Future research should focus on the improvement of the electrochemical setup used. The modular bioelectrochemical device presented allows for the performance of different electrode structures and materials to be tested in electrogenetic systems, such as the various biocompatible carbon-based electrodes that have previously been developed for microbial fuel cells (*59*). Electrochemical limitations (such as mass transfer limitations or redox inducer oxygen reactivity) could also be overcome through the use of quorum sensing systems and positive feedback circuits to propagate gene expression throughout a culture (*20*, *23*). Genomic integration of electrogenetic constructs will also prevent the requirement for antibiotics, which are prone to electrochemical degradation (*60*).

Electrogenetic device performance can also be improved through the identification of redox inducers that exhibit favorable properties, such as appropriate midpoint potentials, high cell permeability, long-term stability, low cytotoxicity, and minimal oxygen reactivity. While inducers can be identified through screening experiments, as was done herein, there remains a limited understanding of the often-complex nature of redox inducer interactions, such as that between methyl viologen and SoxR. Systematic analyses of redox mediators in bioelectrochemical systems have previously been performed to identify how their chemical properties and electron transfer mechanisms can be related to device performance (*27*). Similar analyses of redox inducers in electrogenetic systems could be performed to aid the design of enhanced redox inducers. Identification of stable redox inducers will also enable accurate tuning of gene expression over long time periods through the application of specific working electrode potentials. The use of exogenous redox inducers could be avoided entirely by engineering bacteria to express enzymes that biosynthesize redox inducers; *E. coli* and *Pseudomonas* species have previously been engineered to produce nonendogenous phenazines (*27*, *61*).

The electrogenetic devices presented here can be adapted and integrated for use in a variety of bioelectronics, including medical and environmental biosensors (*4*). The demonstration of electrochemical control of gene expression in relatively large volume cultures (Fig. 6C) also suggests an application in large bioreactors, where low-light penetration prevents the use of optogenetic systems. The SoxR-P*sox*S system is also conserved across a diverse range of bacteria (*62*), with electrochemically inducible gene expression with the system being demonstrated in both *E. coli* and *Salmonella enterica* (*23*), and now *V. natriegens* also. Despite the portability of this system, an expanded electrogenetics toolset made of many different redox-responsive promoter systems and redox inducers would ease the application of this technology. This could also include electrogenetic systems capable of functioning in eukaryotic systems, using redox-sensing transcription factors that have been identified in plants (*63*), animals (*64*), and other eukaryotes. The modular framework for designing electrogenetic systems we have used here (Fig. 1A), as well as our promoter engineering methodology (Figs. 2 and 3), use of genetic logic circuits (Fig. 4), and methods of screening redox inducers (Fig. 5), can all be applied to other redox-sensing regulatory systems in the future to build such a toolset. Furthermore, the use of the BASIC (Biopart Assembly Standard for Idempotent Cloning) assembly standard in this study facilitates easy automation of electrogenetic circuit assembly (*34*), to aid this process. The lack of available tools for constructing electrogenetic systems has severely limited their development. An electrogenetics toolset, of which the tools developed in this paper are an early component, promises to expedite and expand the development of future electrogenetic systems for diverse applications.

## MATERIALS AND METHODS

### Chemicals and reagents

Primers and DNA parts (gBlocks) were synthesized by Integrated DNA Technologies. Polymerase chain reaction (PCR) reactions were carried out using Phusion High-Fidelity DNA Polymerase from Thermo Fisher Scientific. Blunt end ligations were performed using the Thermo Fisher Scientific CloneJET PCR Cloning Kit. Enzyme digestions were performed using high-fidelity restriction enzymes purchased from New England Biolabs (NEB). DNA ligations were performed using Promega T4 ligase. Redox inducers and other chemicals and materials were purchased from Merck, unless otherwise stated. BASIC linkers were obtained from Biolegio, and magnetic DNA purification was done using AMPure XP magnetic DNA purification kits. All organic redox inducers were stored as stock solutions at −20°C: methyl viologen dichloride hydrate (100 mM in LB), pyocyanin [50 mM in dimethyl sulfoxide (DMSO)], DHNA (1 M in DMSO), riboflavin (1 mM in LB), and hydrogen peroxide (1 M in LB).

### Bacterial strains, plasmids, and media

The *E. coli* strains used were DH5α (fhuA2 lacΔU169 phoA glnV44 Φ80′ lacZ ΔM15 gyrA96 recA1 relA1 endA1 thi-1 hsdR17), purchased from NEB, and DJ901 [Δ(argF-lac)169 λ-IN(rrnD-rrnE)1 rpsL179(strR) zjc-2205::Tn10kan Δ(soxS-soxR)566], purchased from the Coli Genetic Stock Center. *E. coli* strains were cultivated in LB or M9 medium and LB-agar plates at 37°C with or without corresponding antibiotics at the following concentrations: kanamycin (50 μg/ml), ampicillin (50 μg/ml), and chloramphenicol (25 μg/ml). *V. natriegens* (WT) strains were cultivated in LBv2 medium and LBv2-agar plates (*46*) with or without chloramphenicol (12.5 μg/ml). All plasmids contain pMB1 origin of replication with either kanamycin or chloramphenicol resistance and mScarlet dropout-cassette at the insertion site. For plate reader experiments shown in fig. S9, cells were grown in M9 medium (1× M9 salts, 0.4% glucose, 2 mM MgSO_4_, and 100 μM CaCl_2_) supplemented with 0.2% casamino acids and 100 mM Mops, as described in (*20*).

### BASIC DNA assembly

All part plasmids were assembled by performing blunt end ligations of gBlocks into pJET1.2/blunt vectors. All construct plasmids were assembled using BASIC assembly (*65*). Parts are listed in table S3, constructs in table S3, and BASIC linkers in table S4. Plasmid maps are available via the Apollo repository (DOI: 10.17863/CAM.78759) or on GitHub (https://github.com/JLawrence96/ElectrogeneticsToolset/tree/DNA). Plasmids are available for order on Addgene (www.addgene.org/Rodrigo_Ledesma-Amaro).

### Mutant library generation

The promoter library was constructed using PCR mutagenesis with Phusion High-Fidelity DNA Polymerase. Divergent 5′ phosphorylated primers carrying the mutations were used to amplify the whole plasmid and introduced the mutations using the standard Phusion DNA polymerase protocol from NEB. Mutagenesis primers are listed in table S5. Reverse mutagenesis primers were used to mutate the −35 site, while forward mutagenesis primers were used to mutate the −10 site. The DNA product was purified using PCR clean-up with QIAquick PCR Purification Kit, followed by Dpn I digestion (NEB) to cut methylated template DNA. Plasmids were religated using Promega T4 before heat shock transformation into *E. coli* DH5α.

### Bacterial transformation

Chemically competent cell stocks of *E. coli* DH5α, DJ901, BL21(DE3), and MDS42 were prepared by the Inoue method (*66*) and stored at −80°C. Heat shock transformation was performed by defrosting 50 μl of competent DH5α cells and immediately adding this to a PCR tube containing 5 μl of plasmid DNA. This mixture was incubated on ice for 20 min before transferring to a thermocycler for heat shock transformation. Heat shock was performed with a protocol consisting of 20 min at 4°C, 45 s at 42°C, and 2 min at 4°C. Following heat shock, 200 μl of SOC broth prewarmed to 37°C was added. Recovery of transformed culture was performed at 37°C for 1 hour before 100 μl was plated on LB agar supplemented with the appropriate antibiotic. Preparation and transformation of electrocompetent *V. natriegens* (WT) was performed according to Tschirhart *et al.* (*46*) using a voltage of 0.8 kV.

### Stationary phase measurements

LB or M9 medium was used for *E. coli* strains and LBv2 medium was used for *V. natriegenes* (WT) throughout. Glycerol stock of strains containing the constructs and control plasmids were streaked on medium-agar, before being incubated overnight at 37°C. Plates were stored at 4°C. Single colonies were picked from these plates and inoculated into 5 ml of medium supplemented with the appropriate antibiotic and incubated overnight at 37°C. Two microliters of overnight cultures were diluted in 198 μl of medium + antibiotics with or without pyocyanin to an optical density at 600 nm (OD_600_) of approximately 0.1. Medium containing pyocyanin was created by diluting pyocyanin stock solutions in medium so that diluted overnight cultures had the desired working concentration of pyocyanin (either 2.5 or 10 μM). These cultures were transferred to wells of a 96-well microplate (Costar), which was sealed with Breathe-Easy sealing membrane that was placed in a Synergy HT microplate reader (BioTek) and incubated at 37°C with orbital shaking at 1000 rpm for 14 hours for *E. coli* and 9 hours for *V. natriegenes* (WT). After incubation, endpoint sfGFP fluorescence (excitation, 485 nm; emission, 528 nm; gain, 40) and OD_600_ measurements were taken with a Synergy HT microplate reader (BioTek). For measuring the upstream activity, RFP measurements (excitation, 590 nm; emission, 645 nm; gain, 70) were also taken. All measurements were taken from the bottom. Outputs were expressed in units of RPU. This was calculated by subtracting blank fluorescence and OD_600_ measurements from the data (which were recorded from measurements of the medium condition without cells), before dividing fluorescence measurements by OD_600_ measurements and expressing them as a ratio relative to the same measurements recorded from the same strain harboring an RPU standard plasmid in the same medium. This plasmid contained the same fluorophore being expressed from a J23101 promoter with the same standardized design used for the other promoters in this study (table S3) (*34*, *37*).

### Response function measurements

Microplates were prepared identically to how they were prepared for stationary phase measurements and, apart from overnight cultures, were diluted in medium + antibiotics containing different redox inducers to a range of different redox inducer concentrations. Response curves were calculated by a previously developed method (*13*) in which experimental data were fitted to a straight line for nonresponsive constructs, to Eq. 1 for Uni-P*sox*S and activator devices, and to Eq. 2 for inverter devices

$$y_{\text{RF}}=y_{0}+(y_{\text{max}}-y_{0})\frac{x^{n}}{K^{n}+x^{n}}$$ (1)

$$y_{\text{RF}}=y_{0}-(y_{0}-y_{\text{min}})\frac{x^{n}}{K^{n}+x^{n}}$$ (2)

where *y*_RF_ is the fitted response function. The fixed parameters were as follows: *x*, the concentration of redox inducer; *y*_0_, the RPU with no redox inducer added; *y*_max_, the largest achieved RPU value across all concentrations of redox inducer. The fitted parameters were as follows: *K*, the sensitivity; *n*, the cooperativity.

Fitting was performed in MATLAB using a custom script using the fminsearch function. Response functions were fit by the least-squares method to minimize the sum of squared estimate of errors (SSE) between the data and the model, as detailed in Eq. 3

$$\text{SSE}=\sum_{i=1}^{N}{\left[y_{\text{RF}}(x_{i})-y_{\text{Ex}}(x_{i})\right]}^{2}$$ (3)

where *y*_RF_(*x_i_*) is the response function RPU value and *y*_Ex_(*x_i_*) is the experimental RPU value for a given concentration of redox inducer *x_i_*, with *N* being the total number of redox inducer concentrations tested.

The dynamic rage was calculated from experimental data, as detailed in Eq. 4 for Uni-P*sox*S and activator devices and in Eq. 5 for inverter devices

$$\text{DynR}=\frac{\;y_{0}}{y_{\text{min}}}$$ (4)

$$\text{DynR}=\frac{\;y_{\text{max}}}{y_{0}}$$ (5)

The *R*^2^ was also calculated for each model to determine the quality of the fit, as detailed in Eq. 6

$$R^{2}=\frac{\text{SSE}}{\sum_{i=1}^{N}{\left[y_{\text{Ex}}(x_{i})-\bar{\;y_{\text{Ex}}(x_{i})}\right]}^{2}}$$ (6)

All experimental and calculated values of response functions are listed in table S1. All code is available via the Apollo repository (DOI: 10.17863/CAM.78759) or on GitHub (https://github.com/JLawrence96/ElectrogeneticsToolset/tree/Code).

### cLogD determination

cLogD values of organic redox inducers were calculated in ChemAxon Marvin using the LogD plugin (*67*). Values were calculated from pH 6.8 to pH 7.8 to three decimal places.

### Cyclic voltammetry

Cyclic voltammetry was performed with a PalmSens EmStat3 Blue. A 20-ml glass vial was used as an electrochemical cell with a glassy carbon working electrode, platinum mesh counter electrode, and a Ag/AgCl reference electrode (fig. S10A). Voltammograms were recorded from 5 ml of LB medium alone or supplemented with 1 mM methyl viologen, 100 μM pyocyanin, or 1 mM DHNA. The cell was heated to 37°C using a hot plate. Three scans were performed for each sample between using a scan rate of 10 mV/s. Scans were recorded both with and without purging. Purging was performed for 30 min with N_2_ gas bubbled through a long needle, with said needle being placed in the headspace of the vial when the scan was recorded to maintain anoxic conditions. The third scan for each experiment was recorded, with the corresponding scan of LB medium over the same potential being subtracted from it. *E*_m_ values were calculated from the purged condition from the potential lying equidistant between the oxidation and reduction peaks. Potentials were converted from millivolts versus Ag/AgCl to millivolts versus SHE by addition of 200 mV.

### Bioelectrochemical device fabrication

The modular bioelectrochemical device used in this study consisted of a stack of acrylic blocks (Engineering & Design Plastics Ltd., UK), PDMS gaskets, carbon paper electrodes (Fuel Cell Store, Texas, US), Nafion 117 membrane, and stainless-steel mesh electrical connectors (MeshDirect Ltd., UK).

From front to back, the stack was formed of solid acrylic blocks (fig. S11A) serving as a front plate, followed by a carbon paper disk working electrode (fig. S11E) with a stainless-steel mesh used as an electrical connector (fig. S11G) and a PDMS gasket (fig. S11B). Then, two acrylic blocks with cylindrical central cavity (fig. S11, C and D) were placed next to each other forming the working chamber. A reference electrode was placed in the working chamber by a vertical port present in one of the acrylic blocks. The working chamber was followed by a disk of Nafion 117 membrane (fig. S11F) sandwiched between two PDMS gasket (fig. S11F). The device continued with an acrylic block with a cylindrical central cavity (fig. S11B) forming the cathodic chamber, followed by a PDMS gasket (fig. S11F), a carbon paper disk counter electrode (fig. S11E), and a stainless-steel mesh used as an electrical connector (fig. S11G). The device was then completed with a solid acrylic block for the back plate (fig. S11A). All those components were fastened together with four stainless-steel screws. The complete device is shown in Fig. 6A and fig. S11H. Magnetic stir bars providing mixing were placed in each chamber, and the device was placed atop a multiplate stirrer (Svelp Scientific, Italy), with rubber stoppers being used to plug any cavities during experiments.

### Electrochemical induction of gene expression

For aerobic activation of gene expression in *E. coli*, overnight cultures were diluted in LB + chloramphenicol to a final OD_600_ of 0.05. Pyocyanin stock was added to cultures to achieve a final concentration of 10 μM. Thirty milliliters of culture was loaded into each chamber of the bioelectrochemical device, which was then sealed with rubber plugs, placed on a magnetic stirrer in an incubator set to 37°C, and connected to a PalmSens EmStat3 Blue. Chronoamperometry was performed for 16 hours with an applied electrode potential of −500 mV versus Ag/AgCl (−300 mV versus SHE) and a sampling rate of 1 s^−1^. Following this, cultures from each chamber were visualized by confocal fluorescence microscopy and concentrated fourfold centrifuging at 3000*g* for 5 min and resuspending the pellet in fresh LB + chloramphenicol medium. OD_600_ was then measured using a Cary 60 ultraviolet-visible (UV-Vis) spectrophotometer, followed by fluorescence measurements using an Edinburgh Instruments FS5 Spectrofluorometer (excitation, 485 nm; excitation bandwidth, 1 nm; emission, 528 nm; emission bandwidth, 0.3 nm). Output was measured by dividing fluorescence measurements by OD_600_ measurements.

For tuning of gene expression in *V. natriegens*, the working chamber of the bioelectrochemical device was loaded with 30 ml of LBv2 + chloramphenicol supplemented with 10 μM pyocyanin and 5 mM potassium ferrocyanide (reduced), while the counter chamber was with 30 ml of LBv2 + chloramphenicol supplemented with 10 μM pyocyanin and 5 mM potassium ferricyanide (oxidized). An Ag/AgCl reference electrode was inserted into the working chamber, and all electrodes were connected to a PalmSens EmStat3 Blue. Both chambers were purged with a gentle stream of N_2_ gas throughout to maintain anaerobic conditions. Following 30 min of purging, the system was preequilibrated by performing chronoamperometry at −500 mV versus Ag/AgCl (−300 mV versus SHE) for 5 min, after which Act106 (*V. natriegens*) was added to the working chamber to a final OD_600_ of 0.3. Following a further 5 min of purging, chronoamperometry was performed with an applied electrode potential of either −500, +250, or +400mV versus Ag/AgCl (−300, +450, or +600 mV versus SHE). Four milliliters of the cell culture was then transferred to a N_2_ gas–flushed Hungate culture tube and incubated in a shaking incubator for 4 hours at 37°C. Cells were then pelleted by centrifugation at 3000*g*, resuspended in 4 ml of fresh LB + chloramphenicol, and bubbled with a gentle stream of compressed air for 30 min while on ice to perform the final oxidation sfGFP to activate its fluorescence (*68*). Cultures were then again pelleted and resuspended in fresh medium, before recording OD_600_ using a Cary 60 UV-Vis Spectrophotometer. Cultures were then diluted to an OD_600_ of 0.5 before recording fluorescence measurements using a Cary Eclipse Fluorescence Spectrometer [excitation, 485 nm; excitation slit, 5 nm; emission, 510 nm; emission slit, 5 nm; PMT (photomultiplier tube) voltage, 700 V]. Output was measured by dividing fluorescence measurements by the OD_600_ of 0.5. Anodic charge was measured by integrating positive regions of the representative chronoamperometry scans.

### Confocal fluorescence microscopy

Live Inv106 (DH5α) cells that were grown in the working and counter chambers of the bioelectrochemical device were immobilized on agarose pads made of 1% low-melt agarose solution in water (*69*). Images were acquired using a Nikon Eclipse Ti wide-field microscope with a Nikon objective lens (Plan APO, 100×/1.45 oil) and a Hamamatsu C11440, ORCA Flash 4.0 camera. Bright-field and fluorescence of sfGFP (λ_excitation_ = 490 nm) images were taken. Images were processed using NIS Elements Viewer and ImageJ software.

## Notes

View/request a protocol for this paper from *Bio-protocol*.
