# Supplementary material for: Synthetic biology and bioelectrochemical tools for electrogenetic system engineering
Source: Sci Adv. 2022 May 4;8(18):eabm5091. doi: 10.1126/sciadv.abm5091 (PMC9067924; doi:10.1126/sciadv.abm5091)
Supplement: Supplementary file 1 — Figs. S1 to S14 Tables S1 to S5 [file sciadv.abm5091_sm.pdf]

Science Advances
AAAS

Supplementary Materials for

Synthetic biology and bioelectrochemical tools for electrogenetic system engineering

Joshua M. Lawrence, Yutong Yin, Paolo Bombelli, Alberto Scarampi, Marko Storch, Laura T. Wey, Alicia Climent-Catala, PixCell iGEM Team, Geoff S. Baldwin, Danny O'Hare, Christopher J. Howe, Jenny Z. Zhang, Thomas E. Ouldridge, Rodrigo Ledesma-Amaro*

*Corresponding author. Email: r.ledesma-amaro@imperial.ac.uk

Published 4 May 2022, *Sci. Adv.* **8**, eabm5091 (2022)
DOI: 10.1126/sciadv.abm5091

This PDF file includes:

Figs. S1 to S14
Tables S1 to S5

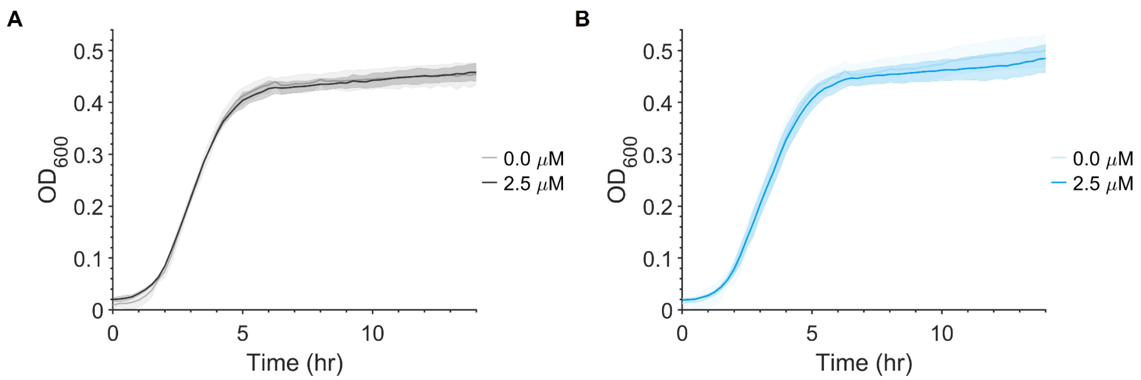

Fig. S1. Cytotoxicity of promoter constructs. (**A**) Negative control (DR) and (**B**) Uni-P*sox*S construct (DR) growth curves with and without 2.5 μM pyocyanin. Solid lines represent the mean from three biological replicates with shaded areas depicting standard deviation of the mean (n = 3).

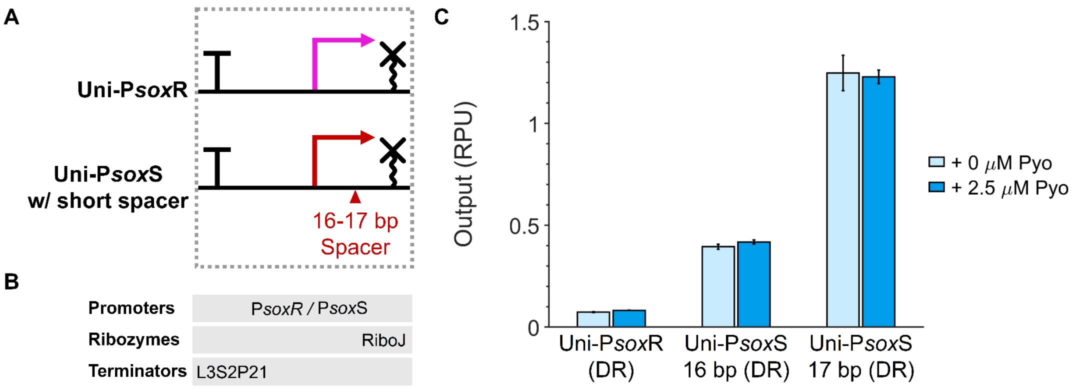

Fig. S2. Screening the activity of alternative P*sox*R and P*sox*S variants. (**A**) Architectures of engineered Uni-P*sox*R promoter and Uni-P*sox*S promoters with shortened spacers. Promoters were tested using the genetic circuit in Fig. 2C. (**B**) List of genetic parts used. (**C**) Downstream activity of engineered promoters with and without 2.5 μM pyocyanin. Datapoints represent the mean from three biological replicates with error bars depicting standard deviation of the mean (n = 3). DR, downstream reporter; RPU, relative promoter units.

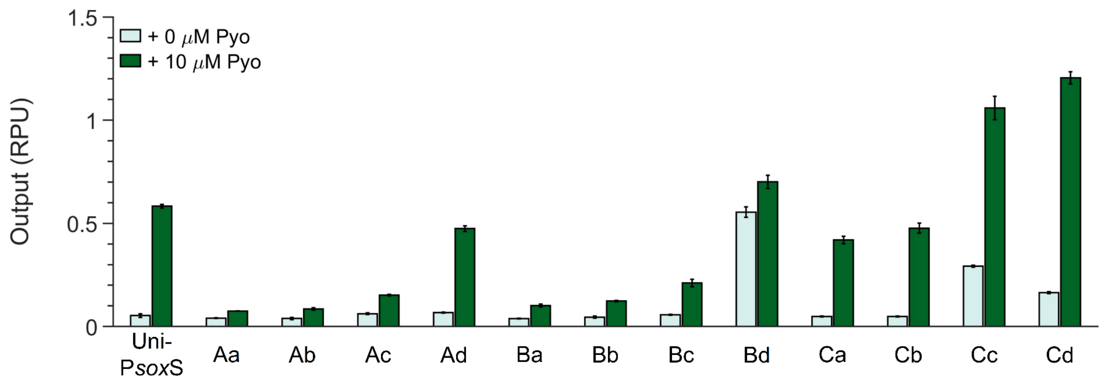

Fig. S3. Screening Uni-P*sox*S promoter library variants. Downstream activity of engineered promoters with and without 2.5 μM pyocyanin. Promoters were tested using the genetic circuit in Fig. 2C. Upper case characters refer to the -35 sites and lowercase characters the -10 sites listed in Fig. 3A. Datapoints represent the mean from three biological replicates with error bars depicting standard deviation of the mean (n = 3). RPU, relative promoter units.

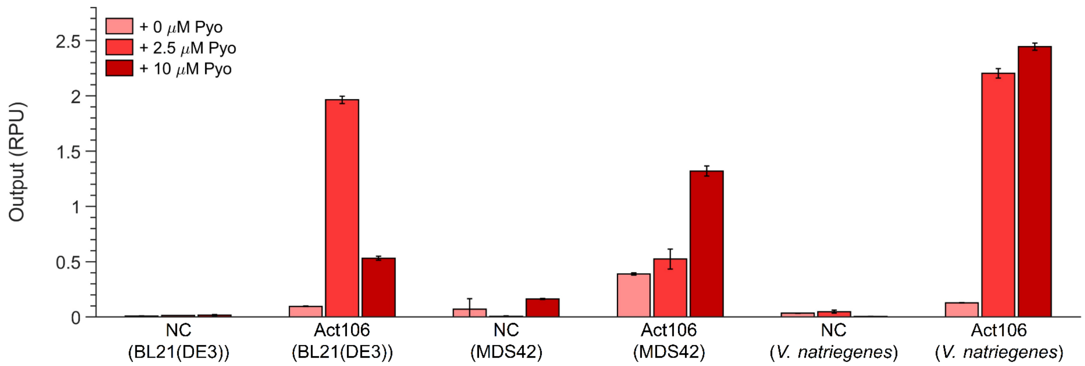

Fig. S4. Screening Electrogenetic Device Performance in Different Bacteria. Activity of activator circuits with and without 2.5 µM and 10 µM pyocyanin in BL21(DE3) and MDS42 strains of *E. coli* and *V.* natriegens (WT). Datapoints represent the mean from three biological replicates with error bars depicting standard deviation (n = 3). NC, negative control.

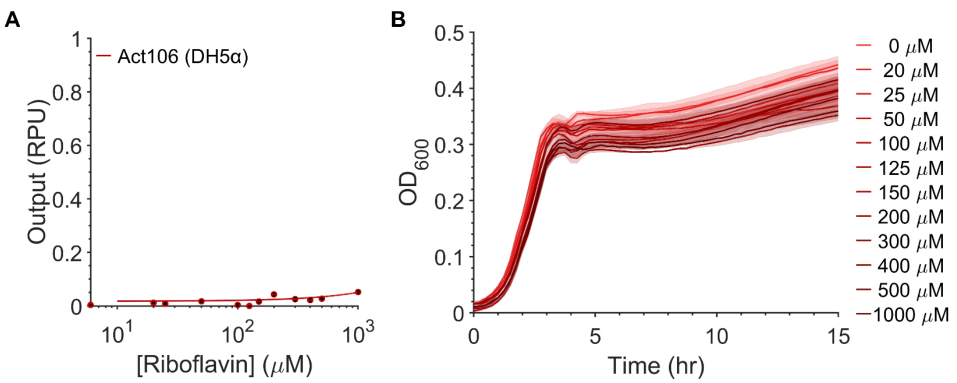

Fig. S5. Electrogenetic circuit performance with riboflavin. (**A**) Response function of Act106 (DJ901) with riboflavin. Datapoints represent the mean from three biological replicates with error bars depicting standard deviation of the mean (n = 3). (**B**) Growth curve of Act106 (DJ901) in increasing concentrations of riboflavin. Solid lines represent the mean from three biological replicates with shaded areas depicting standard deviation of the mean (n = 3).

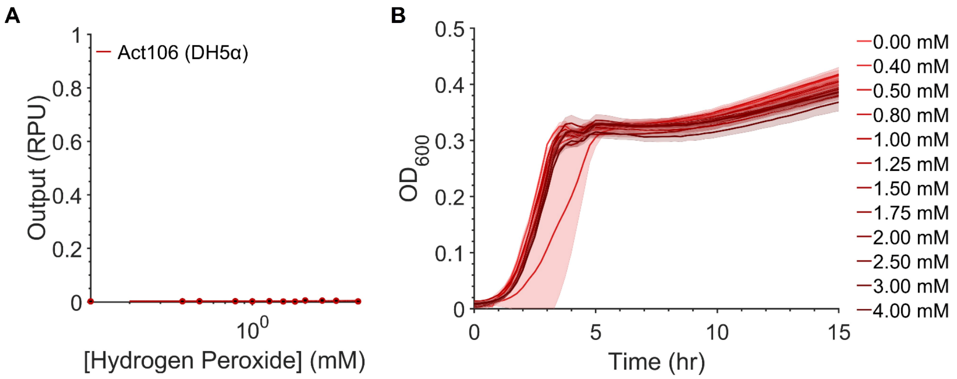

Fig. S6. Electrogenetic circuit performance with hydrogen peroxide. (**A**) Response function of Act106 (DJ901) with hydrogen peroxide. Datapoints represent the mean from three biological replicates with error bars depicting standard deviation of the mean (n = 3). (**B**) Growth curve of Act106 (DJ901) in increasing concentrations of hydrogen peroxide. Solid lines represent the mean from three biological replicates with shaded areas depicting standard deviation of the mean (n = 3).

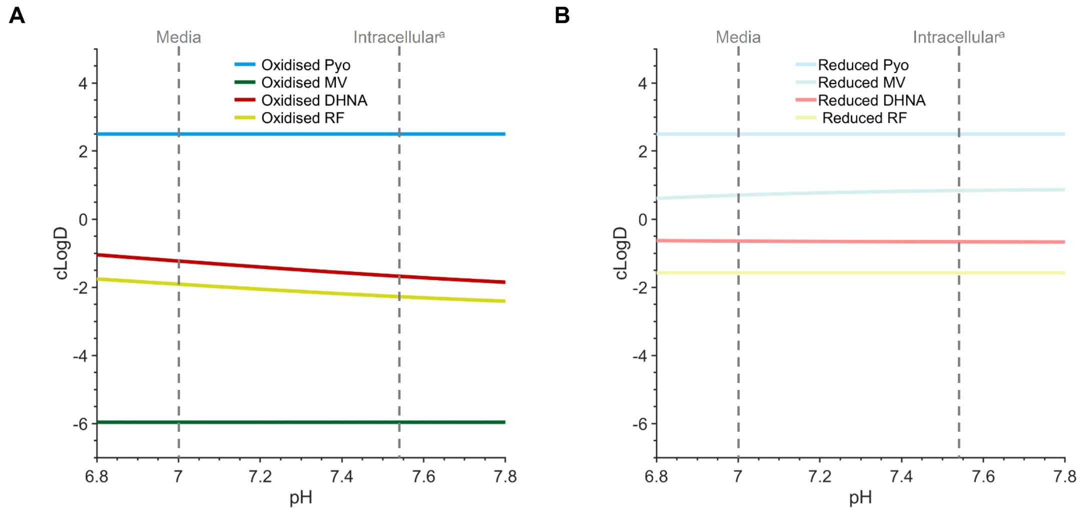

Fig. S7. Predicted Membrane Permeability of Redox Inducers. cLogD of redox inducers in their (**A**) oxidised, and (**B**) reduced state at different pH values. cLogD values were calculated in ChemAxon Marvin(*69*). [a]Intracellular pH was calculated from Slonczewski et al. (*52*). Pyo, Pyocyanin; MV, methyl viologen; RF, riboflavin.

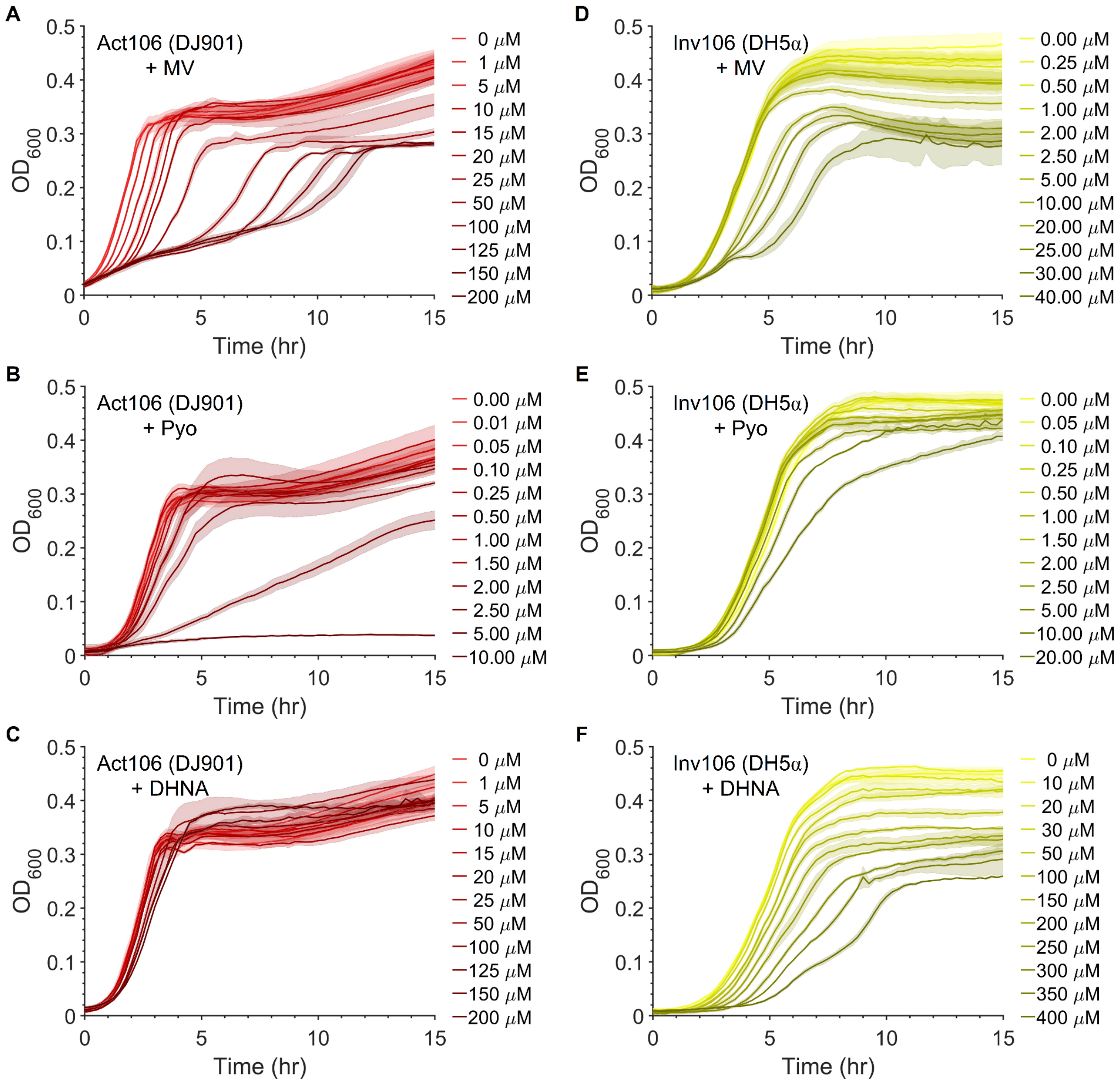

Fig. S8. Cytotoxicity of electrogenetic circuits with diverse redox inducers. (**A-C**) Act106 (DJ901) and (**D-F**) Inv106 (DH5α) growth curves in increasing concentrations of methyl viologen, pyocyanin and DHNA. Solid lines represent the mean from three biological replicates with shaded areas depicting standard deviation of the mean (n = 3).

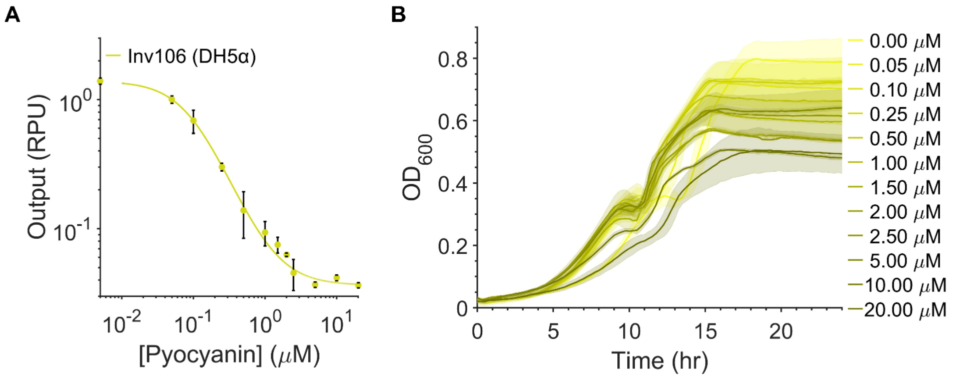

Fig. S9. Electrogenetic circuit performance in minimal media. (**A**) Response function of Inv106 (DH5α) with pyocyanin in M9 media. Datapoints represent the mean from three biological replicates with error bars depicting standard deviation of the mean (n = 3). (**B**) Growth curve of Inv106 (DH5α) in increasing concentrations of pyocyanin in M9 media. Solid lines represent the mean from three biological replicates with shaded areas depicting standard deviation of the mean (n = 3).

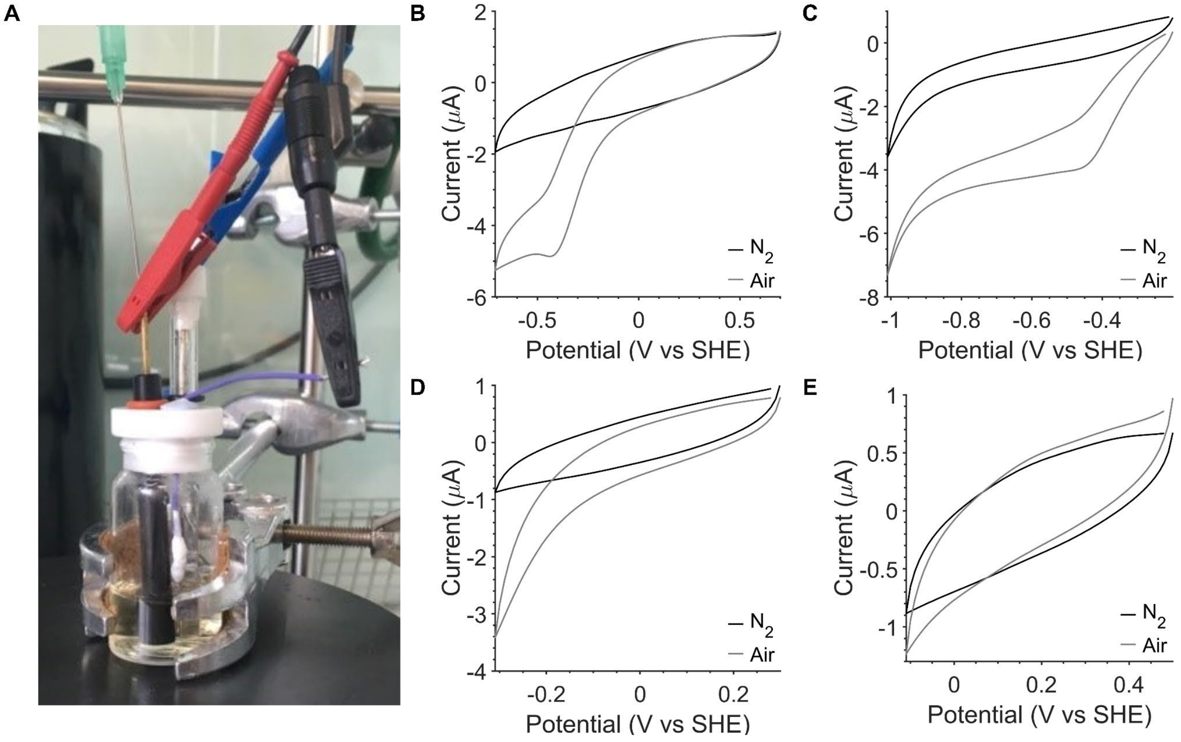

Fig. S10. Cyclic voltammetry of LB media. (**A**) Photograph of the bioelectrochemical cell used for cyclic voltammetry experiments. Red connects to the glassy carbon working electrode, black to the platinum mesh counter electrode and blue to the Ag/AgCl reference electrode. (**B**) Cyclic voltammograms of LB medium over a large potential range. (**C-E**) Cyclic voltammograms performed over the same potential ranges as Fig. 5B-D. The third scan of each voltammogram was recorded using a scan rate of 10 mV s^{-1}. Voltammograms were recorded both in air and in electrolyte purged with N_2 gas for 30 mins (to remove oxygen) before measurements were recorded with an N_2 gas stream being maintained in the headspace.

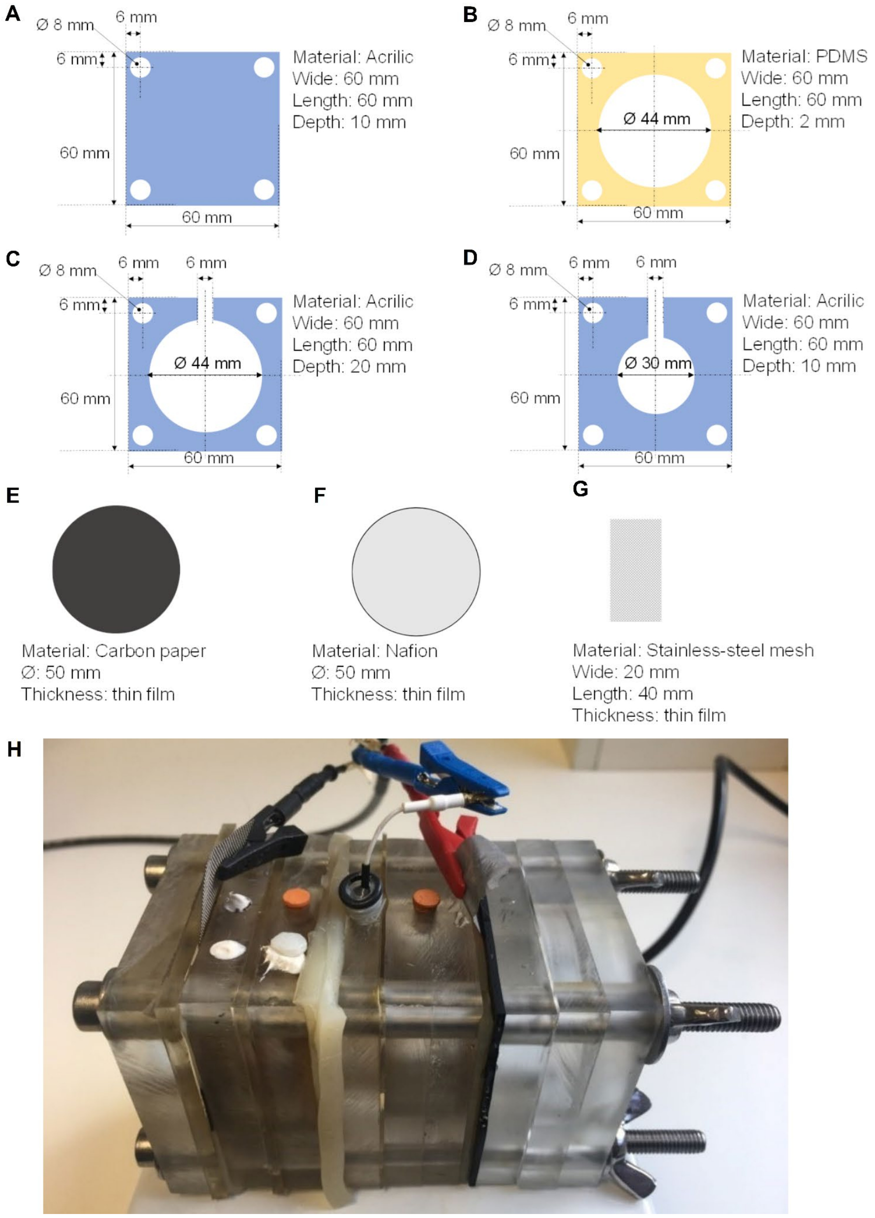

Fig. S11. Bioelectrochemical device design. Dimensions and materials of (**A**) front/back plates, (**B**) gaskets, (**C**) working and counter electrode chambers, (**D**) reference chamber, (**E**) working and counter electrodes, (**F**) Nafion membrane and (**G**) electric connectors. (**H**) Photograph of the assembled device. Red connects to the working electrode, black to the counter electrode and blue to the reference electrode.

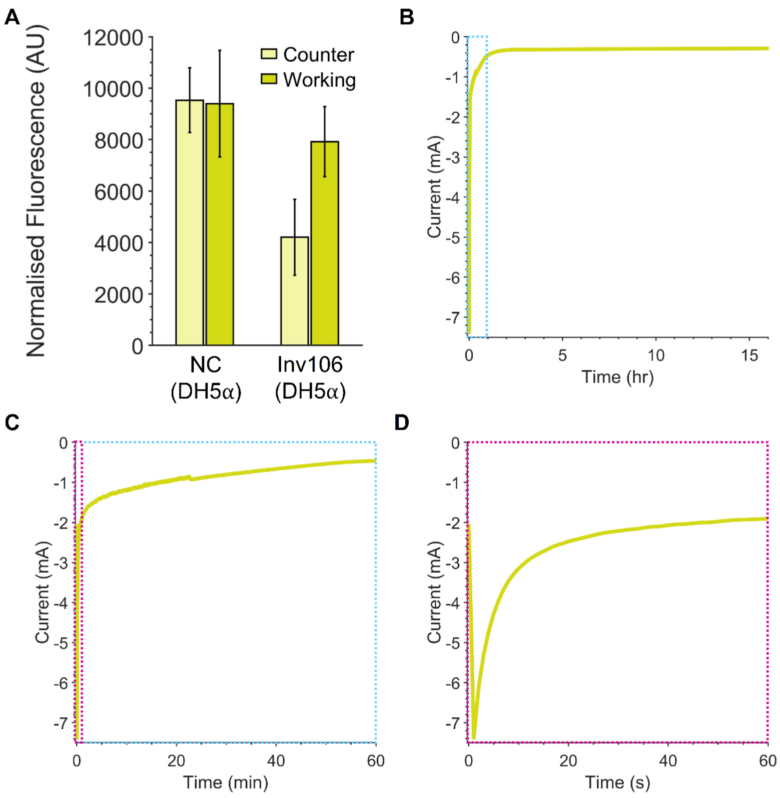

Fig. S12 Chronoamperometry of Inv106 (DH5α) in the bioelectrochemical device. (**A**) Gene expression change in cells between the working and counter chambers measured by cell-normalised fluorescence. Datapoints represent the mean from three biological replicates with error bars depicting standard deviation of the mean (n = 3). (**B**) Representative chronoamperometric scan recorded during electrochemical activation of gene expression of an Inv106 (DH5α) culture in LB medium supplemented with chloramphenicol and 10 µM pyocyanin. Scans were performed over 16 hours with an applied bias potential of -300 mV vs SHE at the working electrode and a sampling rate of 1 s^{-1}. (**C-D**) Zoom in of the first hour and first minute of the scan.

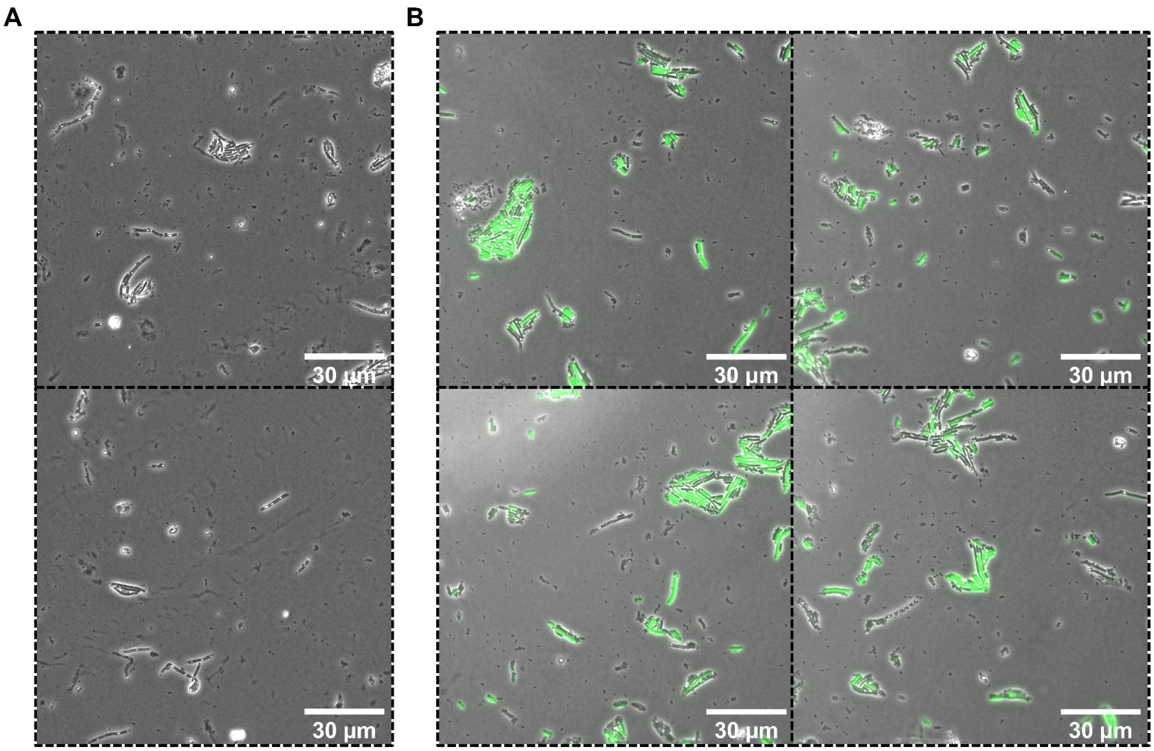

Fig. S13. Imaging electrochemical activation of gene expression. Uncropped confocal fluorescence micrographs of Inv106 (DH5α) grown in the (**A**) counter electrode and (**B**) working electrode chamber. sfGFP fluorescence in green is overlaid on brightfield images. All micrographs were recorded from a single biological replicate.

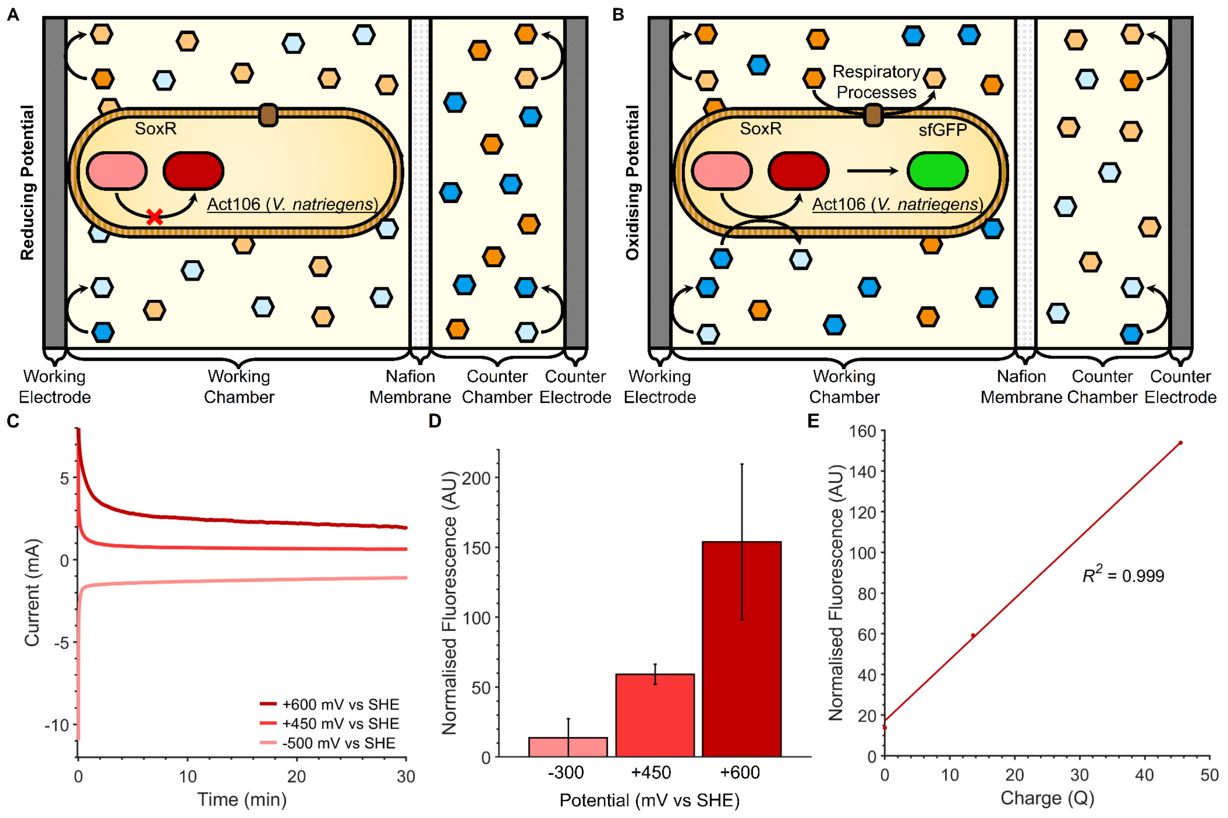

Fig. S14. Electrochemical Tuning of Gene Expression in *V. natriegens*. (**A-B**) Schematic of device operation in reducing and oxidising conditions. With the application of a reducing potential, pyocyanin and ferrocyanide are maintained in a reduced state, preventing oxidation of SoxR in Act106 (*V. natriegens*) cells. With the application of an oxidising potential, pyocyanin and ferricyanide are oxidised, thereby allowing for oxidation of SoxR and activation of sfGFP expression. The electrochemical reaction in the working chamber is driven by an opposite reaction occurring in the counter chamber without cells. (**C**) Representative chronoamperometric scans recorded during application of a working electrode potential of either -300, +450 or +600 mV vs SHE to an Act106 (*V. natriegens*) culture in LBv2 media supplemented with chloramphenicol, 10 µM pyocyanin and 5 mM ferrocyanide. Scans were performed over 30 minutes with a sampling rate of 0.1 s^{-1}. (**D**) Gene expression in cells following the application of different working electrode potentials measured by cell-normalised fluorescence. Datapoints represent the mean from three biological replicates with error bars depicting standard deviation of the mean (n = 3). (**E**) Correlation between the anodic charge calculated from the representative chronoamperometric scans and the mean normalised fluorescence values. An R^2 of the fit is labelled.

Construct	Inducer	Max Concentration (μM)	y_0 (RPU)	y_{min}/y_{max} (RPU)	$DynR$	n	K (μM)	R^2
Uni-P*sox*S	Pyocyanin	40	0.079	0.884	11.19	1.22	4.29	0.979
Act106 (DJ901)	Methyl Viologen	200	0.105	2.492	23.68	1.04	22.12	0.892
Inv106 (DH5α)	Methyl Viologen	40	0.839	0.053	15.70	1.70	4.76	0.996
Act106 (DJ901)	Pyocyanin	10	0.105	4.824	46.06	1.88	1.46	0.998
Inv106 (DH5α)	Pyocyanin	20	0.996	0.020	49.97	1.37	0.57	0.998
Act106 (DJ901)	DHNA	200	0.099	2.116	21.39	2.57	64.38	0.977
Inv106 (DH5α)	DHNA	400	0.977	0.248	3.93	2.55	138.82	0.991
Inv106 (DH5α)	Pyocyanin (M9)	20	0.289	0.008	38.15	1.44	0.09	1.000

Table S1. Response function parameters.

Plasmid Index	Name	Function	Antibiotic	Part Length (bp)
pPEG.000	pBR322-BASIC Kan^R	Construct Acceptor	Kanamycin	3698
pPEG.001	pBR322-BASIC Cm^R	Construct Acceptor	Chloramphenicol	3554
pPEG.100	Native P*sox*R/S	Promoter	Ampicillin	63
pPEG.101	Bi-P*sox*S	Promoter	Ampicillin	219
pPEG.102	Uni-P*sox*S	Promoter	Ampicillin	209
pPEG.103	Uni-P*sox*S-Aa	Promoter	Ampicillin	209
pPEG.104	Uni-P*sox*S-Ab	Promoter	Ampicillin	209
pPEG.105	Uni-P*sox*S-Ac	Promoter	Ampicillin	209
pPEG.106	Uni-P*sox*S-Ad	Promoter	Ampicillin	209
pPEG.107	Uni-P*sox*S-Ba	Promoter	Ampicillin	209
pPEG.108	Uni-P*sox*S-Bb	Promoter	Ampicillin	209
pPEG.109	Uni-P*sox*S-Bc	Promoter	Ampicillin	209
pPEG.110	Uni-P*sox*S-Bd	Promoter	Ampicillin	209
pPEG.111	Uni-P*sox*S-Ca	Promoter	Ampicillin	209
pPEG.112	Uni-P*sox*S-Cb	Promoter	Ampicillin	209
pPEG.113	Uni-P*sox*S-Cc	Promoter	Ampicillin	209
pPEG.114	Uni-P*sox*S-Cd	Promoter	Ampicillin	209
pPEG.115	Uni-P*sox*R	Promoter	Ampicillin	208
pPEG.116	Uni-P*sox*S 16 bp	Promoter	Ampicillin	206
pPEG.117	Uni-P*sox*S 17 bp	Promoter	Ampicillin	207
pPEG.118	J23101	Promoter	Ampicillin	191
pPEG.119	J23101_Inverted	Promoter	Ampicillin	191
pPEG.120	J23105	Promoter	Ampicillin	187
pPEG.121	J23106	Promoter	Ampicillin	191
pPEG.122	P*phl*F	Promoter	Ampicillin	238
pPEG.200	GFP	CDS	Ampicillin	714
pPEG.201	RFP_Inverted	RBS+CDS+Terminator	Ampicillin	925
pPEG.202	SoxR	CDS	Ampicillin	465
pPEG.203	PhlF	CDS	Ampicillin	603
pPEG.300	B14	Terminator	Ampicillin	95

Table S2. BASIC parts list. Full plasmid maps are contained within the supplementary files.

Plasmid Index	Name	Backbone	Antibiotic	Parts & Linkers
pCEG.000K	NC (Promoter)	pPEG.000	Kanamycin	LMP - pPEG.300 - LMS
pCEG.000C	NC (Act)	pPEG.001	Chloramphenicol	LMP - pPEG.300 - LMS
pCEG.001	Native (DR + UR)	pPEG.000	Kanamycin	LMP - pPEG.201 - L4 - pPEG.100 - U2RBS3 - pPEG.200 - LMS
pCEG.002	Native (DR)	pPEG.000	Kanamycin	LMP - pPEG.100 - U2RBS3 - pPEG.200 - LMS
pCEG.003	Bi-P*sox*S (DR + UR)	pPEG.000	Kanamycin	LMP - pPEG.201 - L4 - pPEG.101 - U2RBS3 - pPEG.200 - LMS
pCEG.004	Bi-P*sox* (DR)	pPEG.000	Kanamycin	LMP - pPEG.101 - U2RBS3 - pPEG.200 - LMS
pCEG.005	Uni-P*sox*S (DR + UR)	pPEG.000	Kanamycin	LMP - pPEG.201 - L4 - pPEG.102 - U2RBS3 - pPEG.200 - LMS
pCEG.006	Uni-P*sox*S (DR)	pPEG.000	Kanamycin	LMP - pPEG.102 - U2RBS3 - pPEG.200 - LMS
pCEG.007	Uni-P*sox*R (DR)	pPEG.000	Kanamycin	LMP - pPEG.115 - U2RBS3 - pPEG.200 - LMS
pCEG.008	Uni-P*sox*S 16 bp (DR)	pPEG.000	Kanamycin	LMP - pPEG.116 - U2RBS3 - pPEG.200 - LMS
pCEG.009	Uni-P*sox*S 17 bp (DR)	pPEG.000	Kanamycin	LMP - pPEG.117 - U2RBS3 - pPEG.200 - LMS
pCEG.010	Act105	pPEG.001	Chloramphenicol	LMP - pPEG.120 - U3RBS2 - pPEG.202 - L4 - pPEG.102 - U2RBS3 - pPEG.200 - LMS
pCEG.011	Act106	pPEG.001	Chloramphenicol	LMP - pPEG.121 - U3RBS2 - pPEG.202 - L4 - pPEG.102 - U2RBS3 - pPEG.200 - LMS
pCEG.012	NC (Inv)	pPEG.001	Chloramphenicol	LMP - pPEG.122 - U1RBS3 - pPEG.200 - L1 - pPEG.300 - L2 - pPEG.118 - U3RBS1 - pPEG.203 - LMS
pCEG.013	Inv105	pPEG.001	Chloramphenicol	LMP - pPEG.122 - U1RBS3 - pPEG.200 - L1 - pPEG.300 - L2 - pPEG.102 - U3RBS1 - pPEG.203 - L4 -pPEG.120 - U2RBS2 - pPEG.202 - LMS
pCEG.014	Inv106	pPEG.001	Chloramphenicol	LMP - pPEG.122 - U1RBS3 - pPEG.200 - L1 - pPEG.300 - L2 - pPEG.102 - U3RBS1 - pPEG.203 - L4 -pPEG.121 - U2RBS2 - pPEG.202 -
pCEG.015	RPU_Calibrant (GFP)	pPEG.000	Kanamycin	LMP - pPEG.118 - U2RBS3 - pPEG.200 - LMS

Table S3. BASIC construct list. Parts are in red, standard BASIC linkers in pink, methylated prefix/suffix linkers in blue and RBS linkers in orange. Full plasmid maps are contained within the supplementary files.

Name	Linker Type	Sequence (5'-3')
L1	Neutral	CTCGttacttacgacaCTCCGAGACAGTCAGAGGGTAtttattgaactaGTCC
L2	Neutral	CTCGatcggtgtgaaaAGTCAGTATCCAGTCGTGTAGttcttattacctGTCC
L4	Neutral	CTCGagaagtagtgccACAGACAGTATTGCTTACGAGttgatttatcctGTCC
LMP	Methylated	CTCGggtaagaactcgCACTTCGTGGAAACACTATTATCtggtgggtctctGTCC
LMS	Methylated	CTCGggagacctatcgGTAATAACAGTCCAATCTGGTGTaacttcggaatcGTCC
U1RBS3	RBS	CTCGttgaacaccgtcTCAGGTAAGTATCAGTTGTAAAAagaggagaaataGTCC
U2RBS2	RBS	CTCGtgttactattggCTGAGATAAGGGTAGCAGAAAAAagaggggaaataGTCC
U2RBS3	RBS	CTCGtgttactattggCTGAGATAAGGGTAGCAGAAAAAagaggagaaataGTCC
U3RBS1	RBS	CTCGgtatctcgtggtCTGACGGTAAAATCTATTGTAATcacacaggactaGTCC
U3RBS2	RBS	CTCGgtatctcgtggtCTGACGGTAAAATCTATTGTAAAagaggggaaataGTCC

Table S4. BASIC linker list. Sequences are annealed linker sequences after BASIC assembly. Scar regions are in red and upper case. Overhang regions are in black and upper case and adapter regions are in pink and lower case, unless they have other functionality. Prefix and suffix regions of methylated linkers are in blue and RBSs in orange.

Name	Orientation	Function	Sequence (5’-3’)
SP1_pJET_F	Forward	Sequencing	CGACTCACTATAGGGAGAGCGGC
SP2_pJET_R	Reverse	Sequencing	AAGAACATCGATTTTCCATGGCAG
SP3_pBR322_F	Forward	Sequencing	GGCGGCGGATTTGTCCTAC
SP4_pBR322_R	Reverse	Sequencing	GGACCCCTGGATTCTCACC
SP5_RFP_F	Forward	Sequencing	GGCTCGGGAGACCTATCGATCTT
SP6_RFP_R	Reverse	Sequencing	GCCATCTAGTATTTCTCCTCTTT
CP1_Psox_F	Forward	Cloning	TCTGGTGGGTCTCTGTCCAAATCGCTTTACCTCAAGTTAACTT
CP2_Psox_R	Reverse	Cloning	CGATAGGTCTCCCGAGCCCAGTTCGTTAATTCATCTGTTGG
CP3_SoxR_F	Forward	Cloning	TCTGGTGGGTCTCTGTCCTTAGTTTTGTTCATCTTCCAGCA
CP4_SoxR_R	Reverse	Cloning	CGATAGGTCTCCCGAGCCATGGAAAAGAAATTACCCCG
MP1_35A	Reverse	Mutagenesis	Pi-TAACTTGAGGTAAAGCGATTTAGATTTTCCAGAGTAGGCAC
MP2_35B	Reverse	Mutagenesis	Pi-TAACTTGAGGGCAAGCGATTTAGATTTTCCAGAGTAGGCAC
MP3_35C	Reverse	Mutagenesis	Pi-TAACTTGAGGTCAAGCGATTTAGATTTTCCAGAGTAGGCAC
MP4_10a	Forward	Mutagenesis	Pi-ACTTGAGGAATTATGTTCCCCAACAGATGAATAGCTG
MP5_10b	Forward	Mutagenesis	Pi-ACTTGAGGAATTATAAACCCCAACAGATGAATAGCTG
MP6_10c	Forward	Mutagenesis	Pi-ACTTGAGGAATAATAATCCCCAACAGATGAATAGCTG
MP7_10d	Forward	Mutagenesis	Pi-ACTTGAGGAATTATAATCCCCAACAGATGAATAGCTG

Table S5. Primer list. Overhang regions are in blue with “Pi” denoting the primers are 5’ phosphorylated.
